# Ningaloo Reef: Shallow Marine Habitats Mapped Using a Hyperspectral Sensor

**DOI:** 10.1371/journal.pone.0070105

**Published:** 2013-07-26

**Authors:** Halina T. Kobryn, Kristin Wouters, Lynnath E. Beckley, Thomas Heege

**Affiliations:** 1 School of Veterinary and Life Sciences, Murdoch University, Murdoch, Western Australia, Australia; 2 EOMAP, Gilching, Germany; Universidade Federal do Rio de Janeiro, Brazil

## Abstract

Research, monitoring and management of large marine protected areas require detailed and up-to-date habitat maps. Ningaloo Marine Park (including the Muiron Islands) in north-western Australia (stretching across three degrees of latitude) was mapped to 20 m depth using HyMap airborne hyperspectral imagery (125 bands) at 3.5 m resolution across the 762 km^2^ of reef environment between the shoreline and reef slope. The imagery was corrected for atmospheric, air-water interface and water column influences to retrieve bottom reflectance and bathymetry using the physics-based Modular Inversion and Processing System. Using field-validated, image-derived spectra from a representative range of cover types, the classification combined a semi-automated, pixel-based approach with fuzzy logic and derivative techniques. Five thematic classification levels for benthic cover (with probability maps) were generated with varying degrees of detail, ranging from a basic one with three classes (biotic, abiotic and mixed) to the most detailed with 46 classes. The latter consisted of all abiotic and biotic seabed components and hard coral growth forms in dominant or mixed states. The overall accuracy of mapping for the most detailed maps was 70% for the highest classification level. Macro-algal communities formed most of the benthic cover, while hard and soft corals represented only about 7% of the mapped area (58.6 km^2^). Dense tabulate coral was the largest coral mosaic type (37% of all corals) and the rest of the corals were a mix of tabulate, digitate, massive and soft corals. Our results show that for this shallow, fringing reef environment situated in the arid tropics, hyperspectral remote sensing techniques can offer an efficient and cost-effective approach to mapping and monitoring reef habitats over large, remote and inaccessible areas.

## Introduction

Coral reefs are complex ecosystems which create diverse habitat mosaics and support a wide range of organisms [1]. Australia has a number of coral reef ecosystems, the largest being the Great Barrier Reef. It also has one of the world’s largest fringing reefs along the Ningaloo coast [1], [2], the longest one on the west coast of any continent [3].

Understanding the complexity of coral reef ecosystems, their monitoring and management require information which includes bathymetry and habitat maps. The Ningaloo region is extensive, stretching across three degrees of latitude (22°–24°S). Access from the shoreline or with small boats is difficult along much of the coast and logistics for field work are significant as the region is remote and there is very limited support infrastructure. Large areas with clear waters such as those off Ningaloo in Western Australia naturally lend themselves to the application of optical remote sensing as a means of gathering data on coral reef habitats.

Habitat maps derived from imagery collected by various remote sensing instruments have become widely used in marine monitoring and management in the past two decades [4]. This has been due to lower costs of remotely sensed data, more user-friendly software, maturing methods for deriving habitat maps and growing awareness by managers and decision makers of the usefulness of these data for conservation, planning, monitoring and management [4]. Definition of marine habitats in this paper follows that of Mumby and Harbourne [5] and incorporates a geomorphic component (abiotic) as well as benthic (biotic) cover.

The current habitat map of Ningaloo Reef includes only general classes based on visual interpretation of aerial photos [6], [7]. It does not cover the Muiron Islands, located to the north of the mainland. Although more detailed visual interpretation of aerial images for benthic habitat mapping incorporating geomorphology was initiated a decade ago for selected northern sections [8], no attempt has been made to generate a detailed habitat map covering the whole area of Ningaloo Reef.

Satellite or airborne remote sensing has increasingly been employed to map coral reef communities worldwide [9]–[16]. While a range of these studies have used high spatial resolution data, e.g. IKONOS [13], [17], [18] or Quickbird [19], most studies using high spectral resolution data have been limited to investigating field spectroscopy rather than airborne hyperspectral data. These studies have demonstrated that, through the use of narrow spectral bands, discrimination is possible between *in situ* hyperspectral reflectance measurements of corals and algae [15], [20]–[22], coral growth forms or species [23]–[28] and healthy and bleached corals [10], [29]. A range of narrow band instruments are capable of subtleties in spectra between specimens, allowing the use of specific absorption features not retrievable from medium resolution multispectral data [14], [27]. Many techniques have been employed in these studies to increase the detection rate of spectral differences between reef components. These have included clustering, principal component and linear discriminant analyses [10], [20], as well as derivative analyses, which highlight differences in the shape of spectral reflectance curves rather than in illumination variations [10], [21], [26], [30].

Only a few studies have attempted mapping of coral reefs using airborne hyperspectral data, such as CASI [31], [32], AAHIS [20], AAHIS and AVIRIS [14] or AISA Eagle [33], though not over large areas. One of the acknowledged drawbacks of existing airborne hyperspectral instruments, and, in fact, most remote sensing imagery for coral reef mapping is their spatial resolution. Even with the high spatial resolution of multispectral sensors such as IKONOS, Quickbird or the hyperspectral CASI, which are able to map at scales of <3 m [34], small patches of most reef substrata are still beyond the resolution of existing remote sensors. Very fine-scale structures in coral reefs cannot be resolved [35] and the heterogeneity and structural complexity leads to problems with mixed pixels as a result of poor spatial resolution [36], [37]. For instance, some studies have concluded that bleached and non-bleached coral colonies would only be distinguishable in pixels of 0.01 m^2^, which currently do not exist in commercial remote sensing instruments [38].

Some authors have acknowledged this mixed pixel factor in coral reefs by creating r„mixture groups to represent various realistic scenarios of change in reef communities„ using *in situ* spectral measurements of different combinations of cover types [26]. Others have used a small number of mixed classes to map coral and non-coral assemblages. Addressing the issue of mixed pixels using currently available airborne and satellite sensors would require classifying not only biologically uniform benthic component/substratum pixels, but also pixels comprising a realistic mix of component types occurring in coral reefs [28], [39].

In this study, using hyperspectral data, we aimed to develop a marine habitat classification system suitable for the entire Ningaloo Reef and to map the seabed habitats. Within the mapped habitats, special attention was given to percentage cover and coral types present. Objectives of the study were to firstly, acquire and compare field and image derived spectra from cover-forming reef components along the Ningaloo Reef. Secondly, we aimed to develop a classification system applicable for the entire reef. Thirdly, we set out to map and extract summaries for the entire reef using an operational approach with standardised processing. The last objective was to test a range of thematic and spatial generalizations relevant for mapping large areas such as Ningaloo Reef.

### Study Area

**Figure 1:**
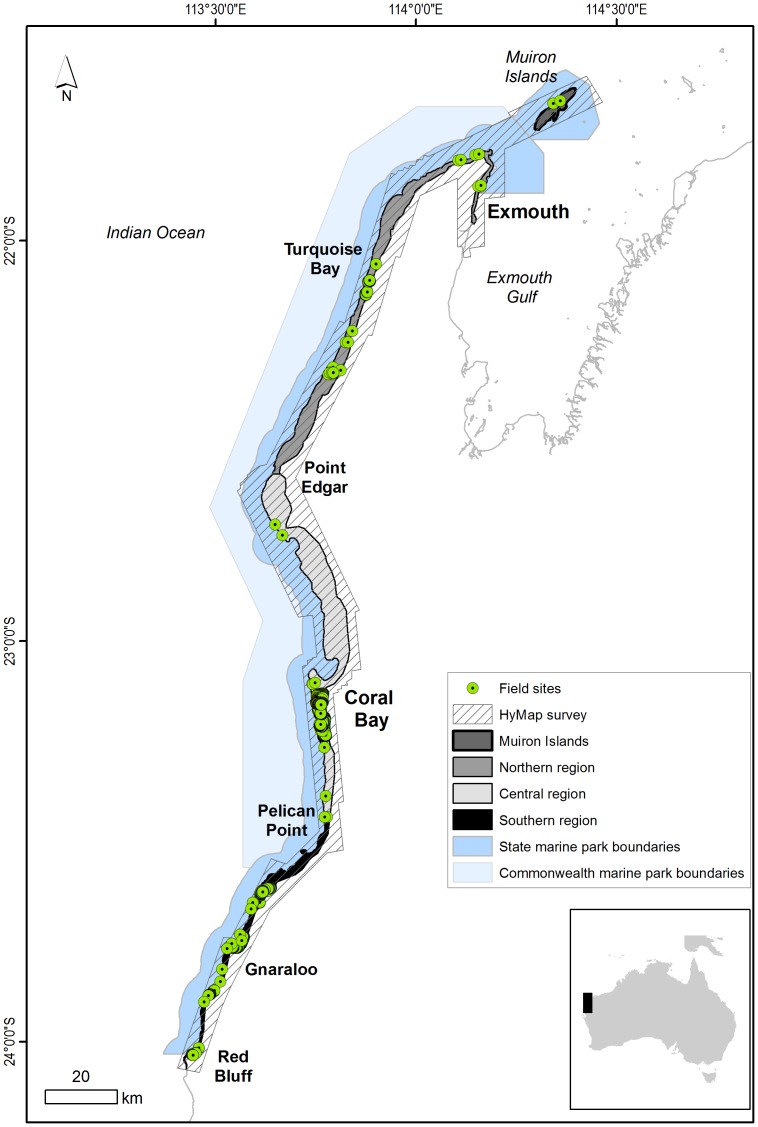
Extent of the HyMap survey area and location of the field sites and key locations at Ningaloo Reef. Outlines of the state and Commonwealth marine park boundaries are also indicated.

Ningaloo is part of the diverse reef system of the Indian Ocean and one of the least anthropogenically disturbed [1]. The reef lies in close proximity to the mainland and stretches over nearly 300 km along the north-west coast of Western Australia (22–24°S) (Figure 1). It is located along one of the narrowest sections of the Australian continental shelf, with the 200 m depth contour less than 20 km offshore. The area lies within the southern Carnarvon Basin geological region and is characterised by limestone features, unstable dune systems, sandy coastal plains and outwash alluvial plains [3]. The modern reef forms a barrier, with occasional passes into the mostly sandy lagoons [1], [2], [8]. Most of the recent reef formations fringe the western shores of the Exmouth Peninsula [1], [40]. Nearly all of the reef area (including the Muiron Islands to the north) has been protected under state and federal legislation [2], [7] and was inscribed on the World Heritage list in June 2011 [41].

The region has a hot and arid climate and the mean annual air temperatures range from 11°C (min) to 38°C (max) [42]. The mean rainfall of the region is approximately 260 mm yr^−1^ and is largely exceeded by the evaporation rate of 1700–3050 mm yr^−1^ [42]. Due to this difference, fluvial run-off is very low and usually only associated with high rainfall, cyclonic events [8]. The sea surface temperatures range between 22–28°C owing to the presence of the southward flowing Leeuwin Current characterized by warm water with relatively low salinity [43]. Very little run-off from the land, as well as the low nutrient and turbidity levels in the ocean, result in clear waters [44], ideally suited for optical remote sensing.

In this study we divided Ningaloo Reef into northern, central and southern regions, based largely on the width of the lagoons and included the Muiron Islands as a separate area since it has not been previously mapped (Figure 1).

## Materials and Methods

### Airborne Data Pre-processing

HyMap data with 125 spectral bands (450–2500 nm range, 26 bands in the visible range) at 15 nm bandwidths and 3.5 m pixels were acquired over 10 days in April and May 2006 by HyVista under contract to the Australian Institute of Marine Science. The total area of the survey covered 3 400 km^2^, encompassing Ningaloo Reef to a 20 m depth, as well as the strip of coastal land adjacent to the Ningaloo Marine Park (Figure 1).

The 67 calibrated sensor radiance flight lines (each approximately 30 km long) were individually processed using the physics-based Modular Inversion and Processing System (MIP) [45]–[47]. Modeling of the azimuthally-resolved, radiative transfer for a multilayer atmosphere - ocean system in MIP is based on the Finite Element Method [48]–[50]. Sunglint correction of the sensor radiance, atmospheric transformation of radiances to the subsurface reflection and the Q-factor correction to account for the bidirectional effects of the water column were performed using the MIP modules (Heege and Fisher [47]).

The resulting flight lines of the subsurface reflectance were geo-referenced and mosaicked to generate 17 image data blocks. The correction of water column-related effects was performed using MIP WATCOR module to retrieve the bathymetry and sea floor reflectance [48], [49] (Figure S1). The transformation of subsurface reflectance to the bottom reflectance was carried out based on the equations by Albert and Mobley [50], assuming approximated constant scattering and absorbing properties of water constituents with up to two different settings of water constituent concentrations per data block. The unknown input value of depth was calculated iteratively in combination with the spectral un-mixing of the respective bottom reflectance. The un-mixing procedure produced the sea floor coverage of three main bottom components (sediment, dark vegetation and bright coral to account for the range of potential albedos) and the residual error between the model bottom reflectance and the calculated reflectance. These specific albedos were extracted from the image in the definition phase of the specific inherent optical properties and then kept constant. The water depth, bottom reflectance, and bottom coverage were calculated at the minimum value of the residual error.

The MIP processing was performed independently of any spectral data collected in the field, such as optical properties of the water constituents, or specific reflectance properties of the sea floor classes. The specific inherent optical properties of the water constituents were first analysed with optical closure calculations in several adjacent deep water areas, and then used as a fixed set of values for the whole reef. All spectra for the sea floor classification were derived by extracting the spectral sea floor characteristics from different sites over the survey area. The statistical variation within each group was analysed for the spectral overlaps between the groups. Class-specific spectral features were used to establish configuration settings for the fuzzy logic discrimination of classes.

### Field Data Collection and Processing

Airborne data processing and development of the image classification and validation data sets were supported by ten field trips to different parts of Ningaloo Reef between 2006 and 2009 as it was not possible to collect all field data around the time of HyMap acquisition. There were several reasons for this, namely, logistics and costs, size of the area, and the airborne data collection schedule which depended on weather conditions made simultaneous collection of field data difficult. However, the majority of data points were collected around the same season (April) with similar macro-algal growth conditions. Data were obtained at locations with fairly homogenous cover type, therefore ensuring their representativeness and allowing for any positional errors [51]. Underwater spectra and site descriptions for a range of cover of uniform and mixed types were collected at a number of sites. Field work was limited by road accessibility to the 300 km long reef, but much effort was made to cover a wide range of ecologically variable areas of the reef (Figure 1).

Spectral reflectance measurements from reef components including sand, coral and algae were collected *in situ* to assess the range of spectral variability within each cover type. Data collection was performed following the methods of [14], [20] using an Ocean Optics USB 2000 portable radiometer. Each site was geo-located with a Garmin GPS unit (accuracy ±5 m). Data on water depth, reef component types and their percentage cover were collected. Analysis of spectral separability of the field spectra was undertaken through the calculation of median, mean, standard deviation as well as first and second derivatives by use of least square (Savitzky – Golay) polynomial smoothing filter of 9 nm width and an order of 3 [52].

### Development of the Classification Hierarchy

As the field data contained information on percentage cover of up to nine cover types per location (Table 1), they were used to create the classification hierarchy and class labels (Figure S2). Three considerations were used to create the classification scheme. Firstly, could the reef components be separated through the *in situ* spectra? Secondly, what was the percentage cover? And thirdly, more emphasis was placed on the development of biotic classes.

**Table 1:** Biotic and abiotic class components at Ningaloo Reef and their codes as used in the field and in the classification system.

Name	Code
Hard coral	HC
Soft coral (e.g. *Sinularia* spp.)	SC
Branching coral	CB
“Blue tip” branching coral (Acropora cervicornis)	CBT
Digitate coral	CD
Encrusting coral	CE
Submassive coral	CS
Tabulate coral	CT
Massive coral	CM
Foliaceous coral	CF
Turfing algae or macro-algae-covered intact dead coral or rubble	TA- or MA-covered IDC or R
Limestone pavement	LP
Macro-algae (consisting largely of *Sargassum myriocystum*)	MA
Rubble	R
Sand	S
Turfing algae	TA

Hierarchy of the habitat classes was based on the type and percentage cover and class labels were made up of a combination of the single cover type in relation to their percentage cover in the sample site. Class selection also incorporated the frequency of occurrence of ground truth points per class in the data set, since a minimum number of points per class was required for training and validation of the classification.

**Figure 2:**
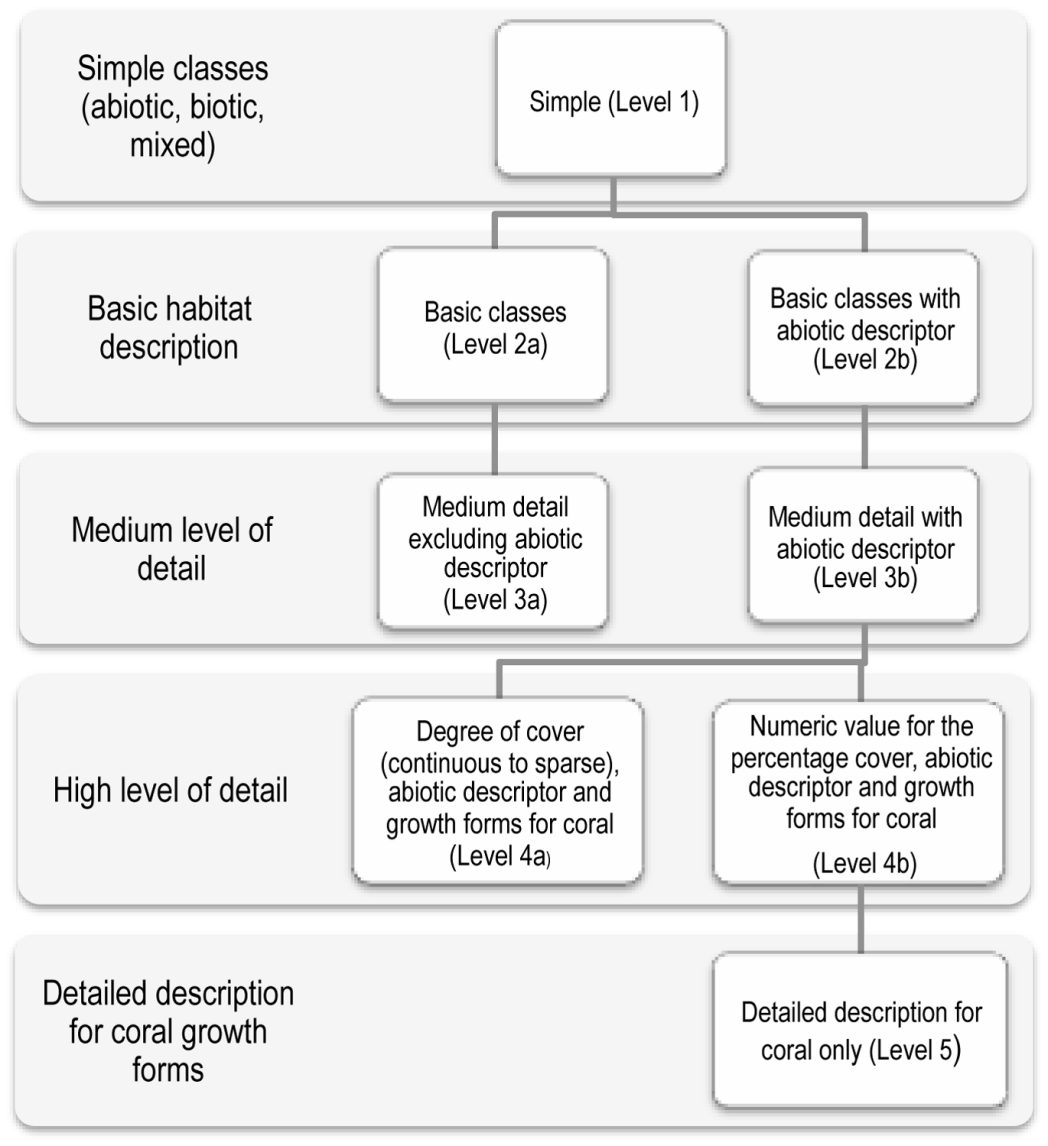
Illustration of the hierarchical system of classification levels used in the Ningaloo Reef marine habitats look-up table.

Spectral analysis of *in situ* spectra was used to determine the merging or exclusion of cover types for the classification. We examined different logical combinations, such as grouping all hard coral, macro-algae and dead coral subcategories to form ‘basic’ classes, each with a percentage cover value as a sum of the subcategories. A number of habitat classes described in the field were aggregated, resulting in the final 16 basic cover types which formed either classes of their own or in various combinations (Table 1). The hierarchy used was based on the percentage cover ranging from 90% or more (continuous cover), 50–90% (dominant category) and two types of mixed classes (<50% cover) (Tables 1 and 2) (Figure 2).

**Table 2:** Summary of the labelling approach for benthic components at Ningaloo Reef using class label and percentage cover.

Class label	Description	Example
Continuous classes	If cover > = 90%, the point was considered as ‘pure’; the remainingcover types were omitted and the class name received a prefix“Continuous”	Component 1 (most dominant) is limestone pavement with 95% cover, component 2 (second most dominant) is hard coral, with 5%, resulting in the label “Continuous limestone pavement” label and component 2 input omitted
Mixed classes with single dominant category	If the dominant type was biotic and cover was between 50–90% andthe difference between the highest and second highest cover> = 30%, the remaining cover types were incorporated into thename. The name was derived from the dominant componentwith the prefix “Dominant”	Component 1 is limestone pavement with 70% cover, component 2 is hard coral with 25% cover, resulting in the label “Dominant limestone pavement” label and the second label of hard coral for component 2
Mixed classes with equal cover	If the (Cover 1– Cover 2) < = 20% they were considered equal;and each received the prefix “equal” if the sum of the equalpercentages > = 90%	Component 1 is limestone pavement with 50% cover, component 2 is hard coral with 45% cover, therefore (Cover1-Cover2< = 20% and component 1 was assigned a label “equal limestone pavement” and component 2 received label “equal hard coral”
Mixed classes that donot fall into the abovecategories	Mixed classes that did not fit into the above categories remainedin the order they were in and receive the prefix “1”, “2”, “3”, etc.depending on their percentage value	Component 1 is limestone pavement with 60% cover; component 2 is hard coral with 35% cover, component 3 is sand with 5% cover, therefore labels were in that order: “1-limestone pavement”, “2-hard coral” and “3-sand”

### Spectral Analysis of Image-derived Spectra

Before the training sites representing final habitat classes could be used for classification, we undertook analysis of image-derived spectra from the 600 field locations. The statistical software package R (R Development Core Team 2008) was used for multivariate spectral analysis to examine class separability, detect outliers and potentially regroup classes. Spectra from the bottom reflectance mosaics were extracted and statistical analyses, including principal component analysis (PCA), hierarchical clustering and Jeffries-Matusita (JM) distance, were performed on the image-derived spectra. A threshold of ≥1.90 was used for JM distance as it indicates good separability [53]. This step allowed for quantification of the spectral separation between class pairs that had previously been identified as spectrally similar in the PCA and cluster analysis.

Following the spectral analysis, the 67 habitat classes established by class frequency analysis were reduced to 46 as a result of deleting classes with high spectral similarity and outlier points. The final spectral library set was randomly stratified to 70% for training and 30% for validation.

### Pixel-based Classification of Cover Types

The habitat mapping was performed on the HyMap mosaics using a supervised classification approach based on the image derived spectral signatures using the MIP software. The classification module incorporated fuzzy logic and first and second order derivatives in addition to reflectance data from the 26 bands which were useable underwater. Rule sets per class included the spectral class ranges as input for the classification. Only one configuration file (spectral signatures) was used in the classification which guaranteed consistent classification results over the whole data set, as well as allowing a more automated and standardised approach. For the final classification, all spectral sub-classes representing sand were merged as an objective of the study was to maximise mapping of classes containing corals. The same rule applied to limestone pavement.

Several post-classification steps were applied to the habitat data, including merging of the image data blocks, masking inconsistencies in deeper areas and generalising the classification at thematic and spatial levels.

### Thematic Generalization and Data Summaries

To facilitate wider access and use of the data, several hierarchical, thematic levels were created for the classification map by generalising and combining the 46 habitat classes in a look-up table, which could be linked to the classification image using GIS software. After extensive consultation with the range of potential users (e.g., ecologists, biologists, conservation managers and planners), the habitat classes were organised at five levels and sub-levels. The logic of the look-up table was from the simplest (most general) to the most detailed (complex) description in terms of the number of reef components, while also allowing capture of the continuum of cover density from very high (continuous >90%) to very sparse (<20%) cover Figure 2.

Class statistics were calculated to determine the distribution, area and percentage cover of marine habitats. They were calculated from the full resolution data to determine the distribution and proportions of the 46 classes across different geographic domains.

### Validation

Accuracy assessment of the classification was performed using field validation points. These were randomly stratified for the classes, so that both frequently and less frequently occurring classes were represented in the validation data in similar proportions. As validation data have the inherent issue of geo-location error either in the imagery or the field data, a radius of 10 m was generated around each validation point and the class labels extracted for pixels within that radius. If the same class as the validation class occurred within the 10 m radius, then the accuracy was accepted as correct.

Due to the high spectral similarity and thus “fuzziness” of classes [54], [55], a fuzzy accuracy assessment approach was selected. This involved accepting accuracy as correct if the validation and classified areas were fuzzy (spectrally similar). Determining which classes were fuzzy was based on the results of the spectral analysis, similarity in cover (i.e. similar amount of cover of same class component could be grouped together), as well as ecological relevance (Table 3). While validation was performed for all thematic levels, for this paper we only present a representative subset for level 4a.

**Table 3:** Examples of accepted class membership in habitat classification data at Ningaloo Reef.

Type of class fuzziness	Examples
Similar degree of cover of one class component	Validation area is class “Sparse macro-algae with sand” and classified area is “Patchy macro-algae with sand”
One or some class component(s) of mixed classes are the sameand other(s) are different	Validation area is class “Patchy macro-algae with sand” and classified area is “Patchy macro-algae with pavement and sand”
Certain coral growth forms spectrally and texturally similar	Validation area is class “Continuous branching coral” and classified area is “Continuous digitate coral”

### Comparison with Previous Ningaloo Habitat Map

The habitat map of the Ningaloo Marine Park, generated by the Department of Environment and Conservation (DEC), includes eight habitat classes: shoreline reef, coral reef community (subtidal), coral reef community (intertidal), macro-algae, subtidal reef (low relief/lagoonal), subtidal reef (low relief/seaward), sand and pelagic. These were created from visual interpretation of aerial photographs [6], [7]. These GIS-based files were clipped to the spatial extent of our mapping and compared by extracting class statistics.

### Ethics Statement

This project was undertaken under the Department of Environment and Conservation permit to enter the Ningaloo Marine Park for the purpose of undertaking research. No live specimens were removed.

## Results

### Spectral Analysis

#### Analysis of *in situ* spectra

**Figure 3:**
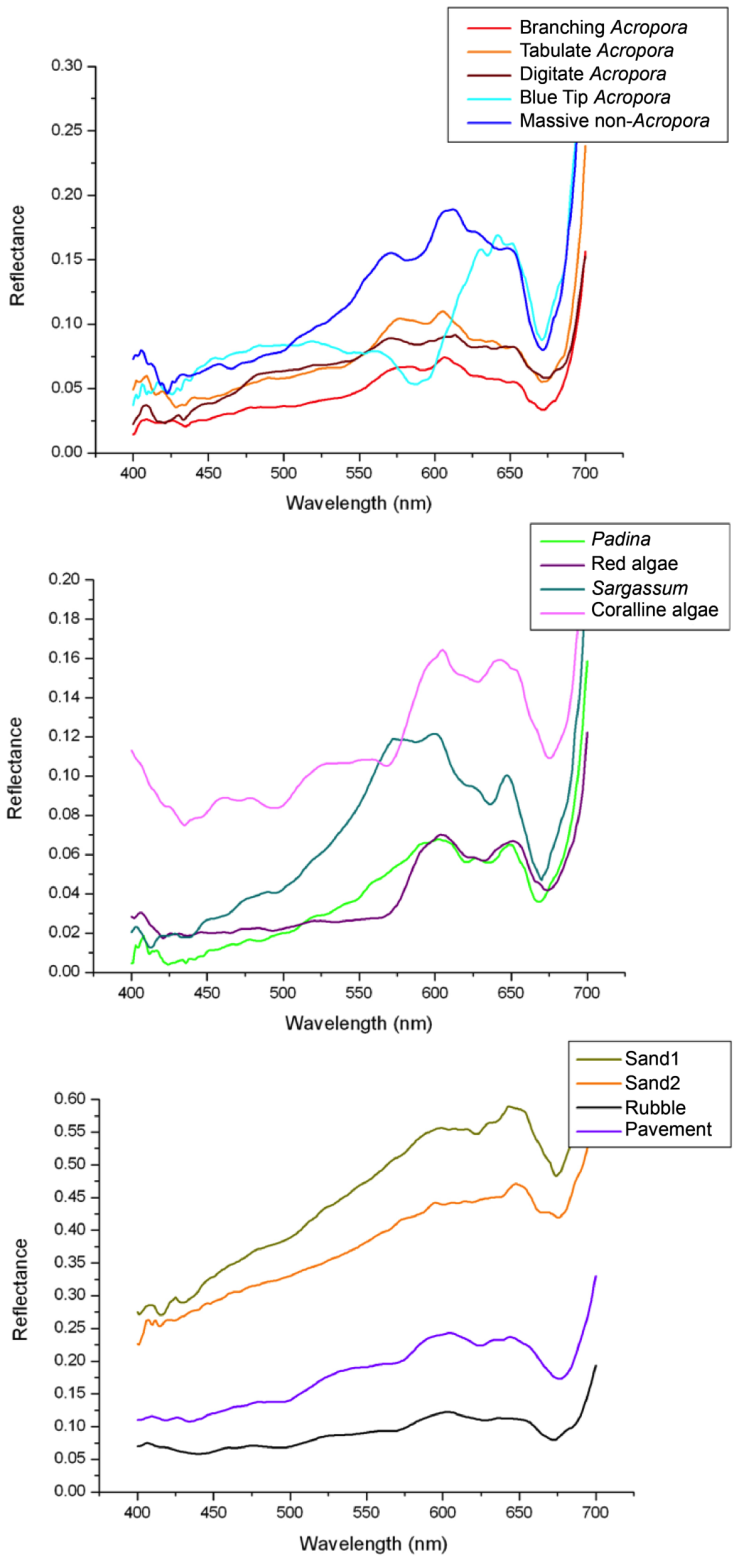
Spectral reflectance (mean values) for selected hard corals (top), macro-algae (centre) and abiotic cover (bottom) at Ningaloo Reef.

Analysis of *in situ* spectra showed clear separation of the main biotic and abiotic habitat components at Ningaloo Reef (Figure 3). The dominant coral genus in the field data was *Acropora* but coral genera were not easily differentiated due to lack of spectral distinction. Instead, corals were separated based on growth forms of branching, digitate and tabulate as well as their colour. All corals showed highest spectral variability in the region between 570–595 nm. Tabulate corals had higher reflectance than branching corals due to texture and shadow effects. Digitate corals had relatively high reflectance, but less defined peaks at 570 nm and 600 nm than tabulate corals. All corals had a reflectance trough at 675 nm (chlorophyll presence). Brown coloured corals, including massive corals showed a triple peak feature at 570, 600 and 650 nm. Distinct “blue-tip” branching coral (*Acropora cervicornis)* had a strong absorption feature at approximately 580 nm with two peaks between 630 nm and 650 nm, and a wide plateau between 450 nm and 520 nm.

At Ningaloo Reef, only frodose macro-algae occurred at spatial scales detectable by the sensor though coralline and turfing algae also occur. *Sargassum* had a strong peak at 570 and 600 nm (similar to brown corals) but with a more defined absorption feature just before the third peak at 650 nm. Non-coralline algae had a reflectance feature at 420–435 nm and between 570–600 nm (Figure 3). Several macro-algae (*Sargassum*, *Padina*, *Dictyota* and *Ulva*) or coralline algae were spectrally separable, but did not occur in large enough patches to be included in the final classification.

Compared to biotic, abiotic cover types had higher reflectance; highest for sand and lowest for dead coral and limestone pavement. Two spectra for sand are presented to illustrate the spectral range (Figure 3). Sand spectra had two peaks at 650 nm and at 600 nm, whereas pavement and dead coral had less pronounced peaks at 600 nm and 630 nm.

#### Analysis of image-derived spectra

As expected, the image-derived spectral analyses of pure reef components covering a whole pixel showed far less differentiation than the *in situ* spectra. This was due to the lower spectral resolution of 15 nm versus 3 nm, as well as the smaller spectral range because of attenuation at longer wavelengths.

**Figure 4:**
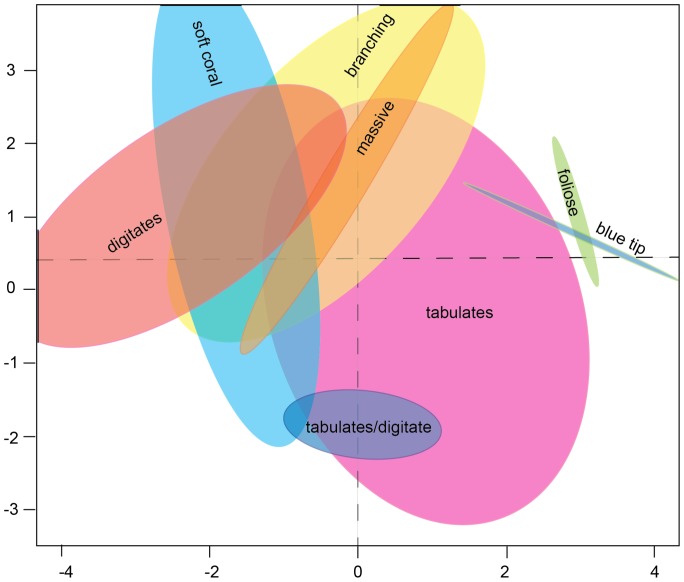
PCA transformed data from image-derived spectra viewed in a two-dimensional space showing continuous (>90% cover) coral classes as well as the tabulate/digitate cluster from Ningaloo Reef. Axes created through the PCA process removed correlations evident in untransformed spectra and allowed identification of outliers, trends and groups.

PCA and hierarchical cluster analysis for the coral classes with more than 90% cover within a pixel showed a high spectral similarity for continuous branching, digitate, massive and soft corals, despite the spectral differences visible in the *in situ* spectral signatures (Figure 3 and Figure 4). Soft corals (Alcyonacea, mostly *Sinularia* spp.) and digitate corals had a JM distance of 1.46 (<1.9, the set threshold), highlighting their similar spectral properties.

PCA and cluster analyses showed that the continuous tabulate coral class had a relatively large spectral range, partly overlapping with the continuous branching and digitate coral classes. However, on applying the JM distance ( = 2) they were found to be separable. Continuous tabulate coral and the mixed class of dominant tabulate coral with digitate coral were not separable with a JM distance of 1.3.

Continuous macro-algae and several mixed coral classes had a high similarity in PCA and cluster analyses but JM distance results indicated that they were separable. Classes that included the same components with a similar degree of cover (e.g., dominant macro-algae or dominant macro-algae with sand <10%) were found to be spectrally similar. Where the biotic cover was sparse or patchy, it had a low spectral influence on the pixel reflectance and the pixel spectrum was driven by the abiotic cover. For example, limestone pavement and sand had large brightness differences, so classes with sparse hard coral (<20%) with limestone pavement or sparse hard coral (<20%) with sand, were differentiated. These results were used to determine the “fuzziness” between classes during the final accuracy assessment.

### Image Classification

**Figure 5:**
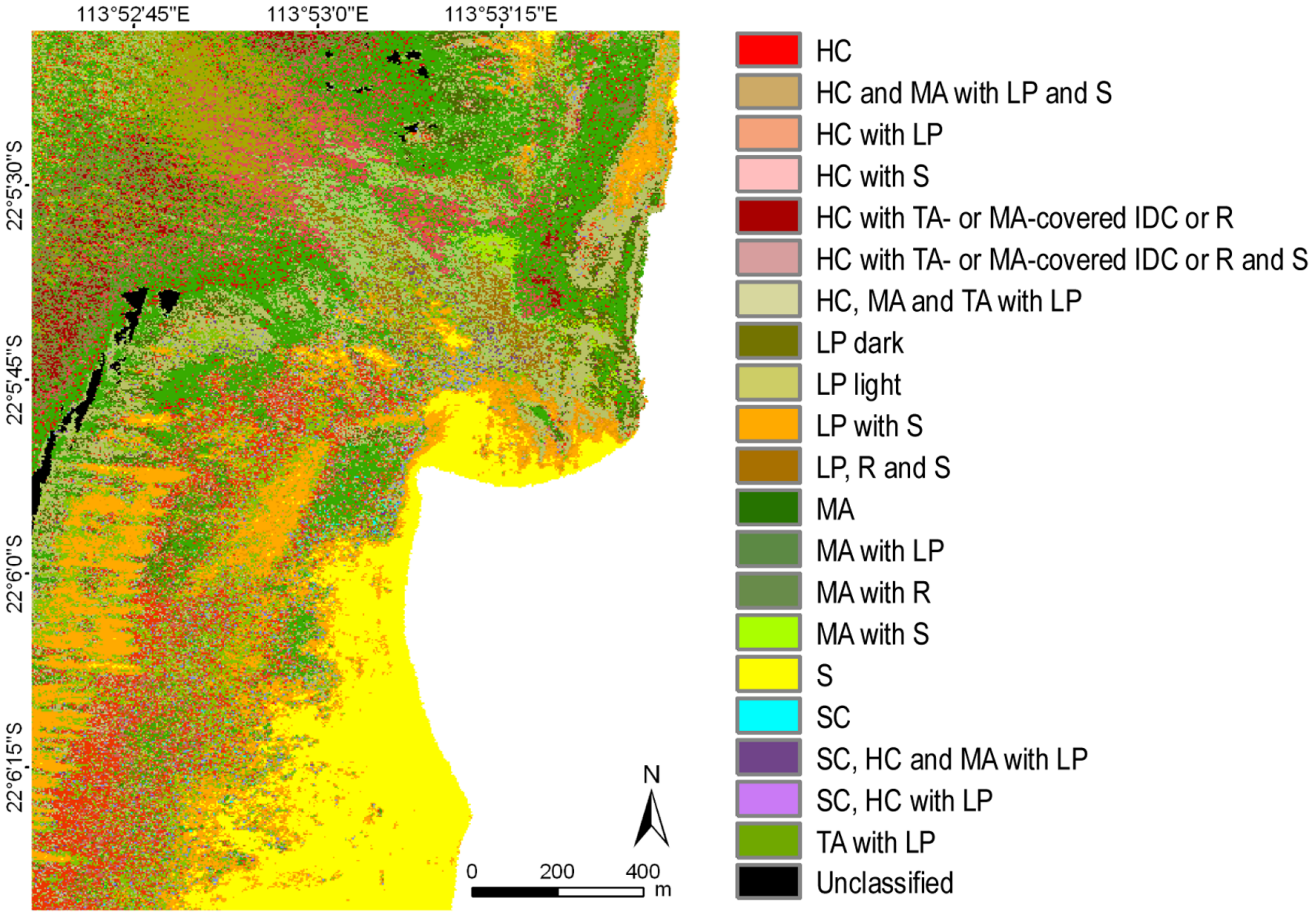
Example of benthic habitats for the Turquoise Bay area of Ningaloo Reef at the thematic classification level 2b. **Legend codes are explained in **Table 1****.****

**Figure 6:**
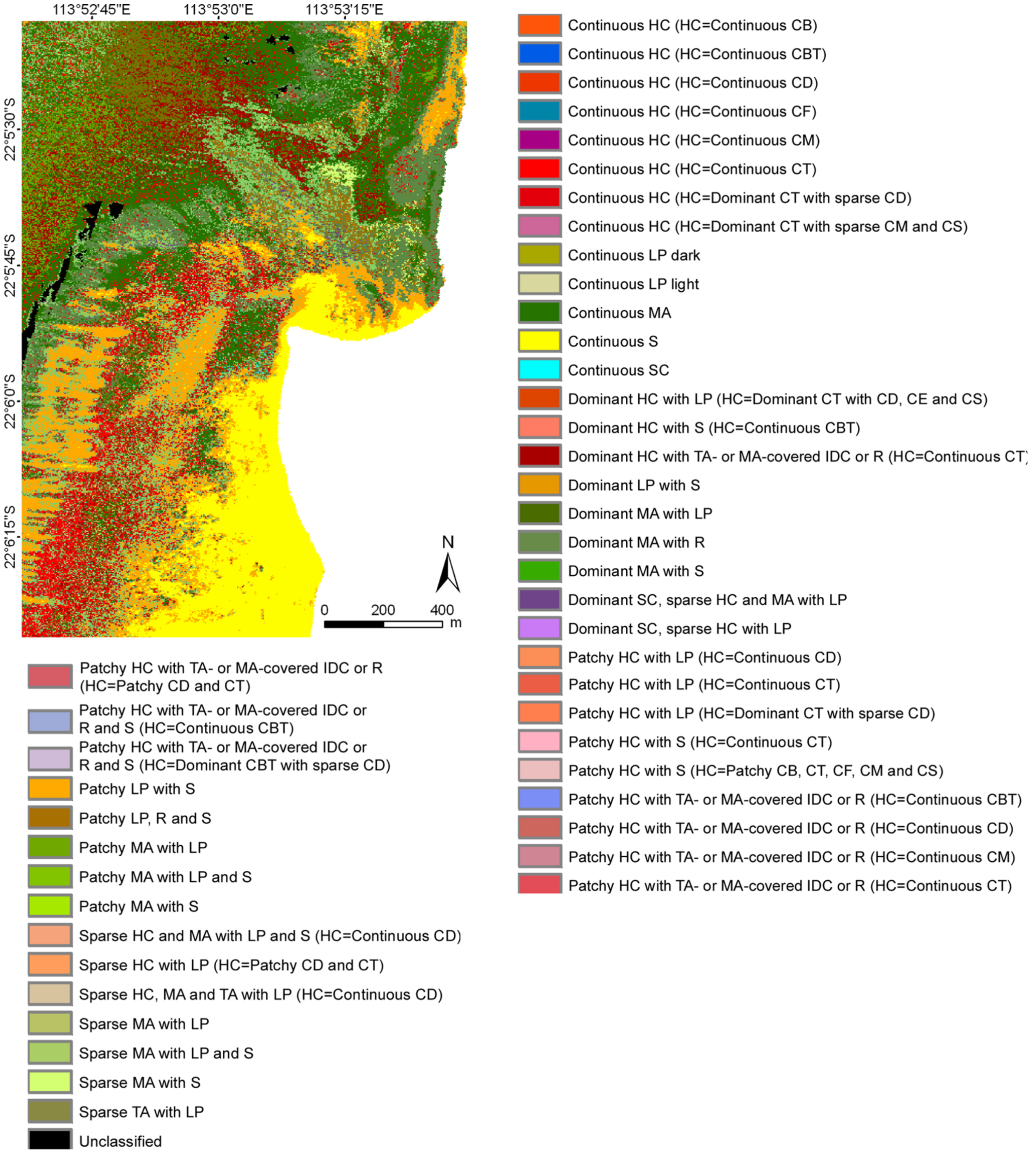
Example of benthic habitats for the Turquoise Bay area of Ningaloo Reef at the thematic classification level 4a. **Legend codes explained in **Table 1****.****

**Figure 7:**
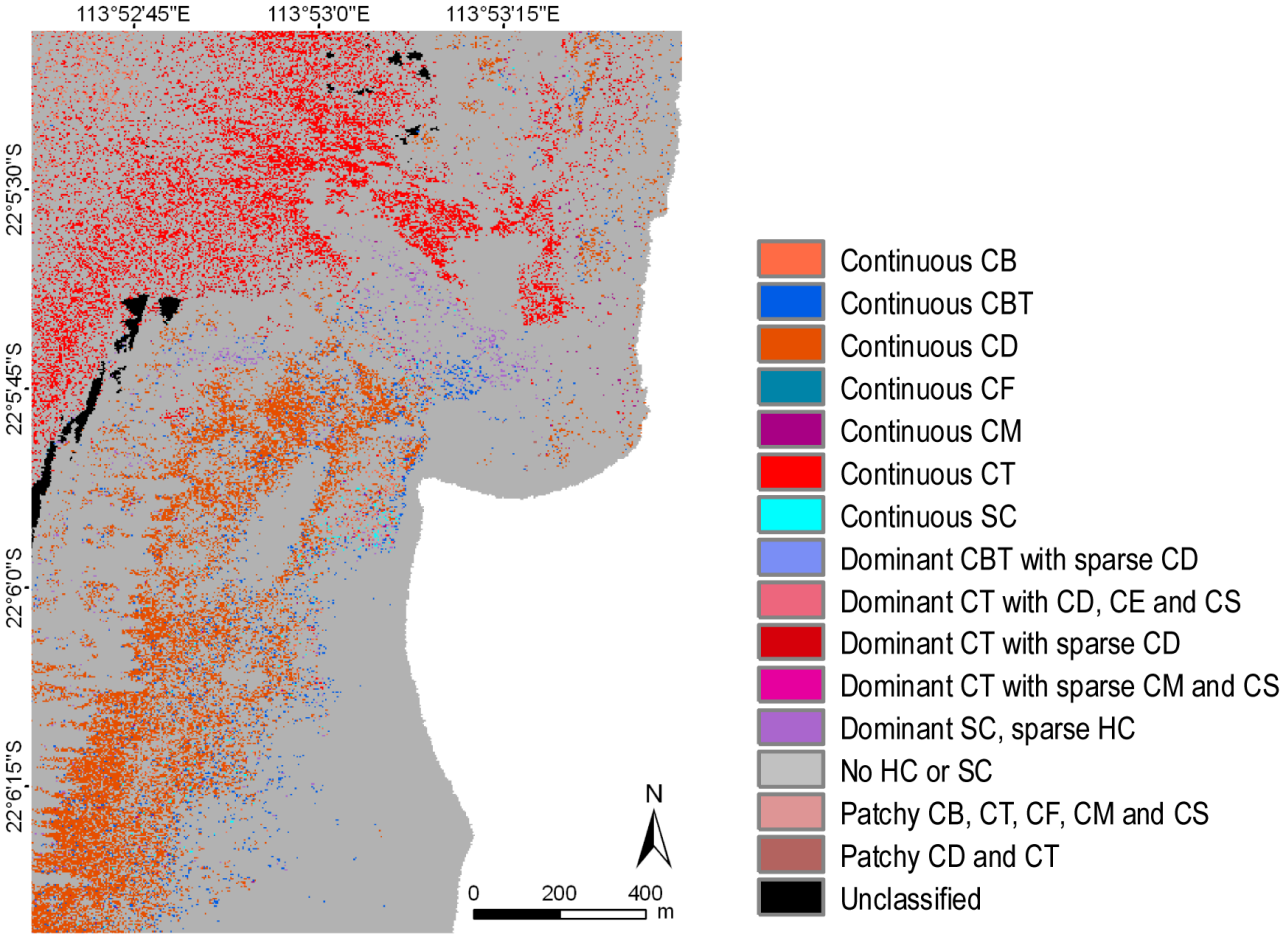
Example of benthic habitats for the Turquoise Bay area of Ningaloo Reef at the thematic classification level 5, showing only information about hard or soft coral classes. Grey areas on the map do not contain any coral component discernible within a pixel. Legend codes explained in Table 1.

The operational approach to classification performed on geo-referenced data mosaics with a single training set and classification parameters for an area that stretched across three degrees of latitude proved very successful. This is especially relevant for large, multi-flight line data sets. Five thematic classification levels and sub-levels were created, ranging from a basic level with three classes (biotic, abiotic and mixed) to the most detailed with 46 habitat classes (consisting of all benthic components and hard coral growth forms in continuous or mixed covers) (Figures 5–7) (Table S1).

#### Regional summary

This study mapped 762 km^2^ of the reef which included 5.9 km^2^ (8%) of coral mosaics (sparse to dense cover), 51% of macro-algae and turfing algae and 41% of sand and limestone pavement (Table 4). Approximately 50% of the areas mapped were in proclaimed sanctuary zones (383 km^2^) and 21% of the mapped area was close to the shore, within 500 m distance from the mean high water mark.

**Table 4:** Summary of areas and percentage components of the main cover types mapped in the four regions at Ningaloo Reef.

Region	Total area mapped (ha)	Classes with corals (ha) (%)	Classes with macro- or turf algae (ha) (%)	Abiotic classes (ha) (%)
Muiron Islands	2419	223 (9%)	1766 (73%)	430 (18%)
Northern	27349	2263 (8%)	14567 (53%)	10519 (39%)
Central	38305	2537 (7%)	17848 (46%)	17920 (47%)
Southern	8090	836 (10%)	4844 (60%)	2410 (30%)
*Total*	*76163*	*5859 (8%)*	*39025 (51%)*	*31279 (41%)*

**Figure 8:**
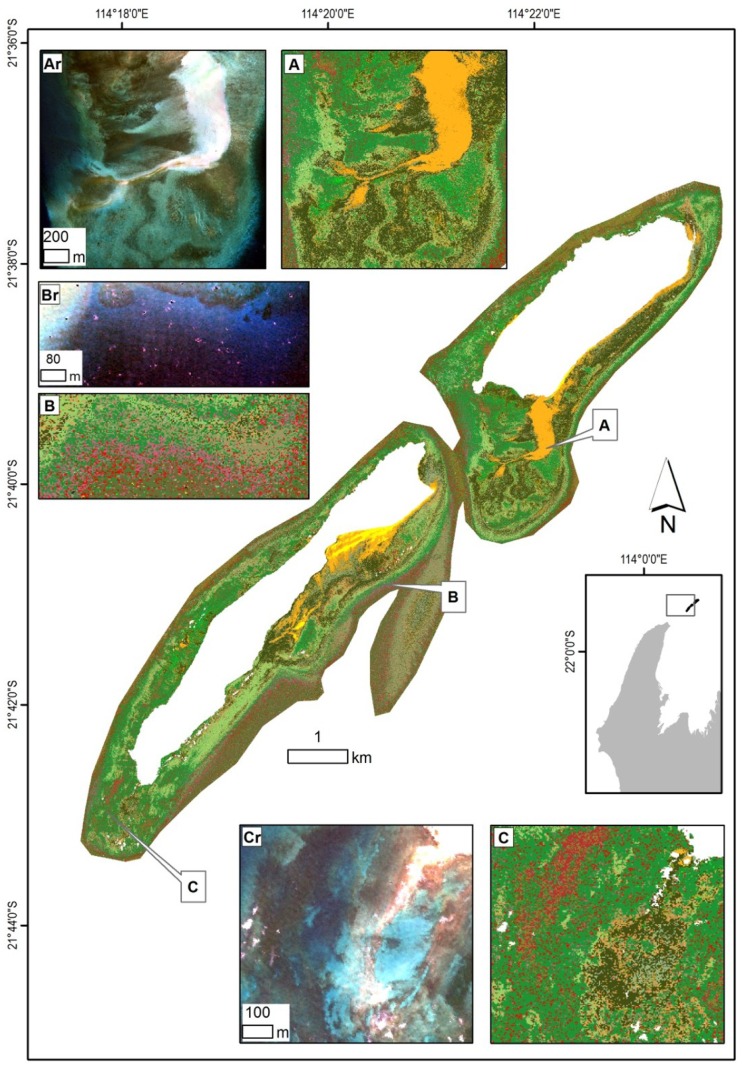
Overview of the Muiron Islands in the northern part of the Ningaloo Reef with insets illustrating selected habitats and corresponding subsurface reflectance. (Ar) Subsurface reflectance of the shallow platform with raised edges and a limestone ridge leading to the Muiron Channel on the left (west), (A) habitats dominated by a limestone platform surrounded by a mix of macro-algae. (Br) Subsurface reflectance of a nearshore area, (B) habitats of the slopes dominated by algae on pavement close to the shore and a zone of dense coral further offshore. (Cr) Subsurface reflectance of a flat limestone platform, (C) habitats with dominant coral cover in the western part and macro-algae with limestone pavement near the shore. Legend from Figure 5 applies.

**Figure 9:**
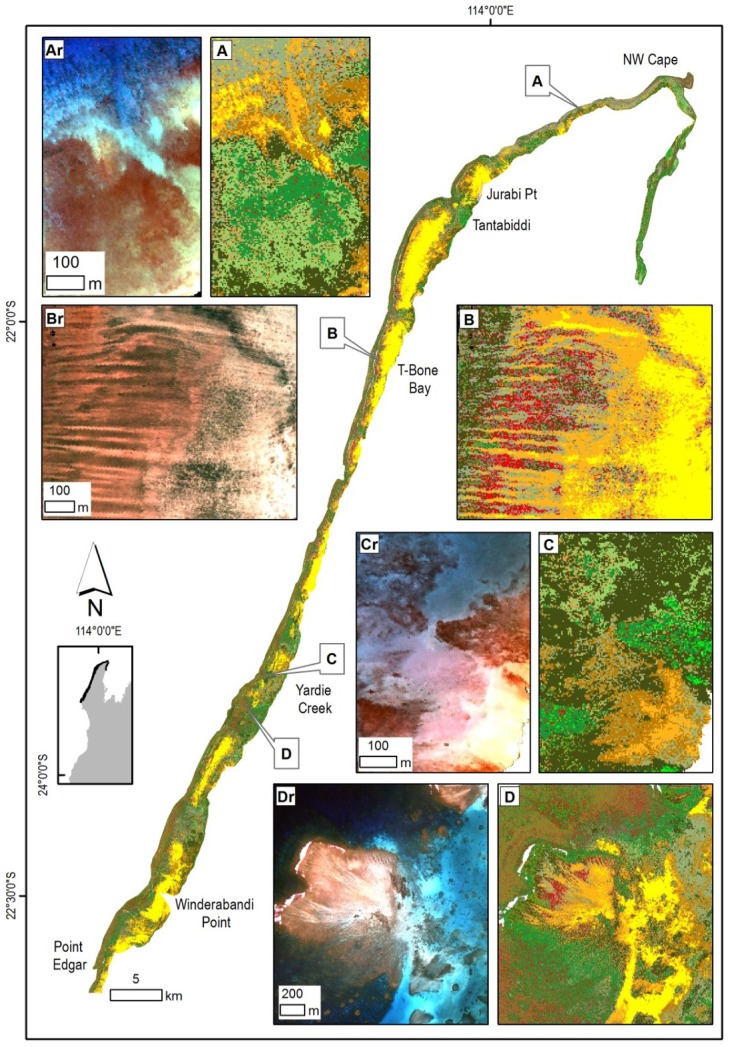
Overview of the northern region of the Ningaloo Reef with insets illustrating selected habitat maps and corresponding subsurface reflectance. (Ar) Subsurface reflectance of nearshore, sublittoral pavement along a rocky shore, (A) habitats of extensive macro-algae, limestone pavement and sand. (Br) Subsurface reflectance of outer reef flat, (B) spur and groove structures with coral and macro-algae transitioning to tabulate coral and sand in the deeper lagoon. (Cr) Subsurface reflectance of the littoral alluvial fan off Yardie Creek, (C) habitats with limestone pavement and adjacent macro-algae with sparse coral. (Dr) Subsurface reflectance of the back reef, (D) back reef on the northern edge of the reef pass with clusters of bommies south and east of the reef flats. Legend from Figure 5 applies.

**Figure 10:**
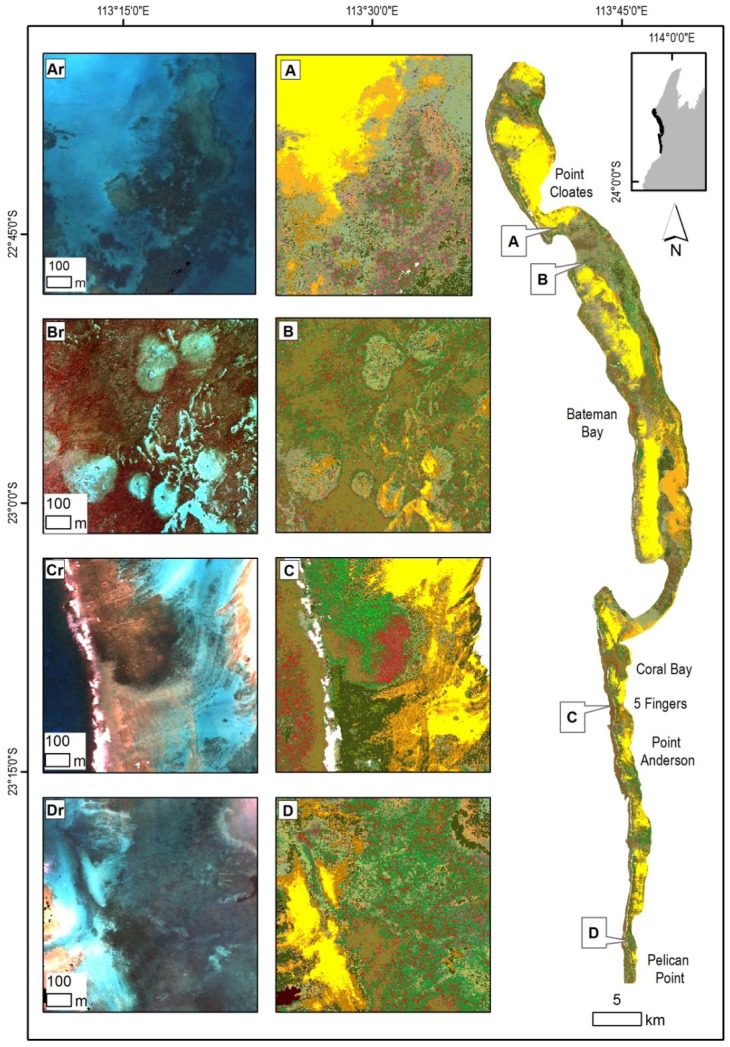
Overview of the central region of the Ningaloo Reef with insets illustrating selected habitat maps and corresponding subsurface reflectance. (Ar) Subsurface reflectance of the edge of the reef flat on the southern edge of a reef pass, (A) habitats of the edge of the reef flat with transition from pavement to patchy macro-algae with a number of coral bommies. (Br) Subsurface reflectance of northern part of a large bay, (B) oval patterns of pavement and sparse macro-algae, with a large bommie in the centre (possibly grazing halos). (Cr) Subsurface reflectance of the Five Fingers Reef, (C) linear limestone ridges with dominant hard coral surrounded by macro-algae due west. (Dr) Subsurface reflectance of a nearshore area south of Pelican Point, (D) complex habitat pattern in the nearshore area with the subtidal platform characterized by pavement, sparse coral, macro-algae and sand. Legend from Figure 5 applies.

**Figure 11:**
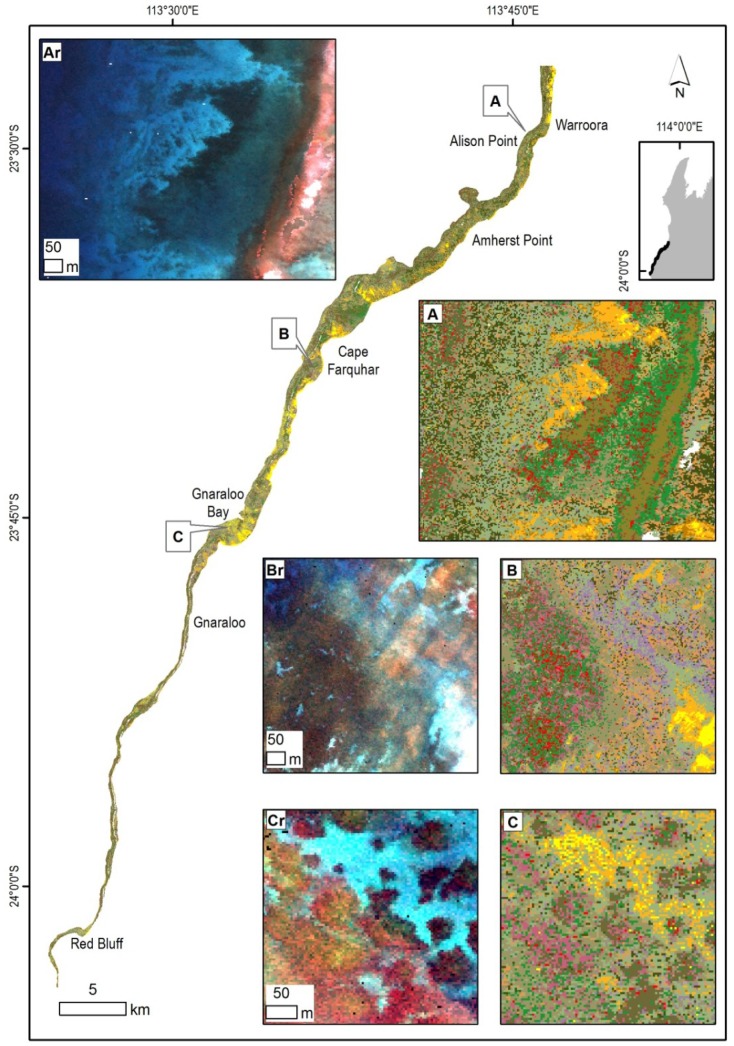
Overview of the southern region of the Ningaloo Reef with insets illustrating selected habitat maps with corresponding subsurface reflectance. (Ar) Subsurface reflectance of a nearshore area, (A) limestone pavement habitats with a long ridge parallel to the shore, covered by patchy to sparse coral and macro-algae. (Br) Subsurface reflectance of an area offshore from Cape Farquhar, (B) dense soft and hard coral cover mosaic with some sand and limestone pavement. (Cr) Subsurface reflectance over a cluster of bommies, (C) habitats dominated by coral bommie clusters on limestone pavement and sand with sparse macro-algae. Legend from Figure 5 applies.

The four regions along the Ningaloo Reef (including the Muiron Islands) showed distinct differences influenced by the bathymetry as well as their geographic position (latitude). The northern and central regions had well developed lagoons, features mostly lacking at the Muiron Islands and in the southern area. The width of the mapped area narrowed down from just over 4 km in the north to about 0.5 km in the south. Four maps (Figures 8–11) which summarise habitat distribution and highlight specific features are presented and use the legend from Figure 5.

The tabulate coral form was the most common, contributing the highest percentage of cover in the Muiron Islands while the lowest percentage cover was in the southern region of the Ningaloo Reef. The coral form which was the second most common varied between the regions; branching and soft corals in the Muiron Islands, digitate in the northern region and soft corals in the central and southern regions.

#### Muiron Islands

The islands have no lagoons and only very narrow platform fringes with widths between 50–300 m then dropping off to depths >10 m. The area mapped was about 24 km^2^ (Table 4). Platforms were wider on the eastern shores with depths <3 m. Habitats were dominated by a combination of macro-algae and limestone pavement classes which made up 90% of the cover (Figure 8). Sparse macro-algae occurred on the slopes. Coral classes comprised 9% of the cover and were found mostly in deeper water (>4 m). The majority of the coral occurred as dense stands of tabulates with >90% cover. Corals occurred in zones abutting limestone pavements, with slightly higher cover at the southern areas of the islands. Some coral was also found as bommies, mostly along the southern shores of the islands (Figure 8).

The two islands are separated by a narrow (0.3 km) channel reaching depths up to 15 m, with limestone, rubble and macro-algae being the main benthic cover. There were also extensive ridges (50–100 m wide) covered by macro-algae, channels and depressions, especially along the western shores.

#### Northern Region

The second largest mapped area, the northern region (Table 4), was quite narrow in the north with 1 km wide shallow areas around Tantabiddi and much wider (up to 4 km) areas in the south near Winderabandi (Figure 9). This region was characterized by a number of wide, shallow (2–5 m depth), sandy lagoons and deeper (up to 20 m) reef passes. Deeper channels corresponded to the drainage pathways from the land (gorges and creek lines). The majority (57%) of the cover was made up by macro-algae, including turfing algae, another 39% with abiotic cover and only 8% of cover contained coral classes.

Over 60% of the coral cover was made up by dense stands of tabulate coral, either nearer the shore or on reef flats; the central lagoons were characterised by sand and limestone pavement. About 10% of coral was the continuous digitate form and a further 7% comprised “blue-tip” branching coral. Soft coral (mostly as 50–85% cover) with up to 20% of digitate or tabular forms with some macro-algae occurred in the southern parts. Massive, submassive and foliose corals were also present, especially as bommies in deeper parts of the lagoons. Approximately 50% of all abiotic classes were made up by sand and another 20% by a combination of limestone with sand. About 6% of abiotic cover consisted of the mixed class containing rubble, pavement and sand. Some 96% of cover of macro-algae was made up by dominant macro-algae with sand, sparse macro-algae with pavement, and patchy macro-algae with sand, and some pavement.

#### Central Region

This region extended from Point Edgar to just south of Pelican Point and covered 383 km^2^. Macro-algae dominated classes were about equal in cover to abiotic classes (Table 4 and Figure 10). Sand and pavement prevailed in the lagoons and some had very large areas of sand (e.g., 11×4 km in Bateman Bay). Coral cover was about 7%. Tabulate coral at very high densities (>90%) was the most common class. Mosaics of tabulate coral with macro-algae or turf algae and rubble made up another 31% of coral cover. Soft coral classes accounted for 8.6% of coral mosaics. Coral was found either inshore as part of the bommie structures (often with massive corals) or closer to reef flats as mixes of tabular, branching and digitate corals. Sparse and patchy macro-algae on limestone and sand were the most common classes, found along 80% of the shoreline and as extensive areas in the northern parts of Bateman Bay (Figure 10).

#### Southern Region

Over half (60%) of the habitats in the relatively narrow coastal strip of 8.1 km^2^ were made up by mosaics of patchy, sparse or dominant macro-algae on pavement and sand. Abiotic classes made up 30% of cover and corals 10% (Table 4 and Figure 11). Six classes made up 95% of the coral cover, with the largest being mosaics of high density soft corals with some tabulate and digitate hard corals. The majority of the coral mosaics occurred as dense stands, with percentage cover between 65–90%.

Macro-algae occurred as mostly patchy or sparse cover (10–45%) on pavement or sand. Abiotic classes were found as either patchy limestone with sand or >90% pavement, with the class containing rubble (with pavement and sand) comprising 18% of cover.

#### Overview of coral classes

**Figure 12:**
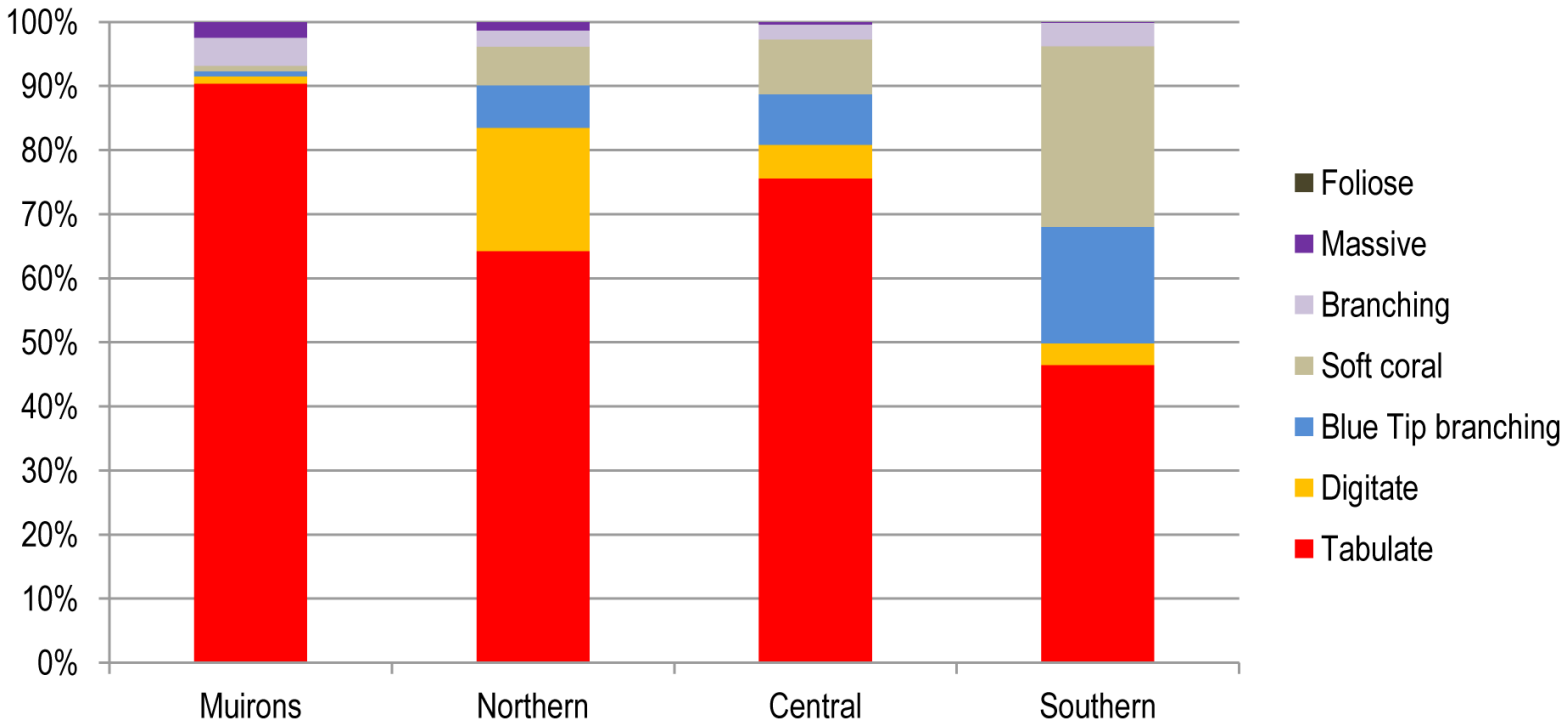
Relative abundance of the main coral forms at Ningaloo Reef by region. This summary incorporates classes with coral cover from 20–100%.

There were 5.9 km^2^ of coral mosaics mapped along the Ningaloo Reef. The single largest coral mosaic was continuous tabulate coral (2.2 km^2^ or 36.7% of all corals) (Figure 12). The majority of the coral classes (66%) were a mix of dense to continuous tabulate coral, sparse digitate coral, soft coral and sparse submassive and massive corals. Continuous to patchy digitate and tabulate coral made up approximately 10% of the coral cover, while “blue tip” *Acropora* was approximately 8.5%. The majority of the hard coral occurred as either very dense (continuous >90%) cover or as patchy distribution (20–45%).

### Probability Images

**Figure 13:**
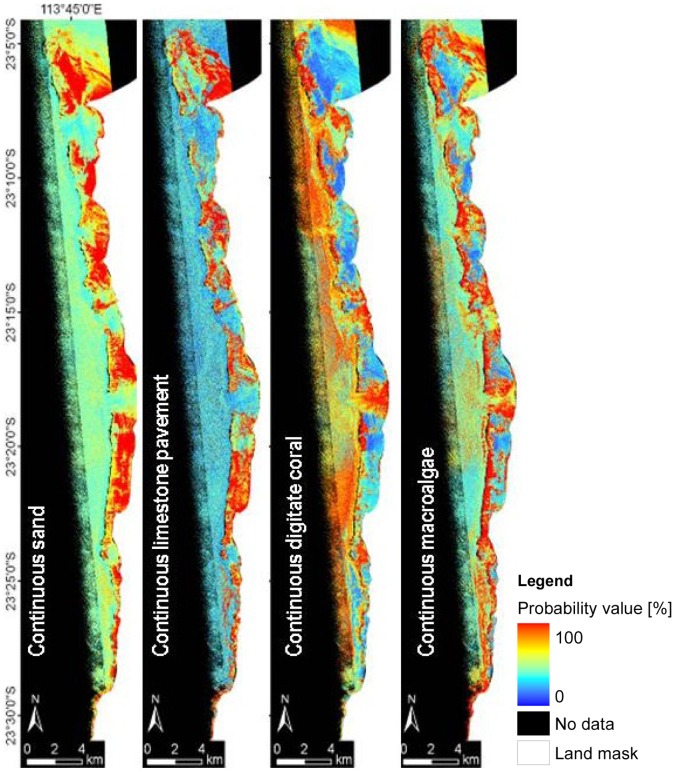
Single class probability images of the Coral Bay area at Ningaloo Reef showing percentage probability for the following classes ‘continuous sand’, ‘continuous limestone pavement’, ‘continuous digitate coral’ and ‘continuous macro-algae’ (from left to right).

Individual per-class probability images were generated during data processing. Examples of single class probability images for four spectrally different classes are shown for the Coral Bay area (Figure 13). Spatial distribution of the class continuous sand can be seen to correspond well to the classification results, with continuous limestone pavement as well as continuous digitate coral and continuous macro-algae having similar probabilities. These probability maps, for example, allow combination of only those classes which include a coral component into a single multi-band file to analyse distribution of coral dominated mosaics.

### Validation Results

The overall accuracy using the 10 m radius and fuzzy logic approach was calculated at 83.81% for level 2a, 70.48% for level 4a with a higher number of fuzzy classes and 63.81% for level 4a with a lower number of fuzzy classes. Validation performed for level 4a showed, as expected, that the higher degree of fuzziness resulted in a higher overall accuracy than the lower degree of fuzziness, with the highest results for level 2a. An example of the confusion matrix at level 4a with high level of fuzziness in class allocation is presented in Table S2.

### Concurrence with Existing Ningaloo Reef Habitat Map

**Figure 14:**
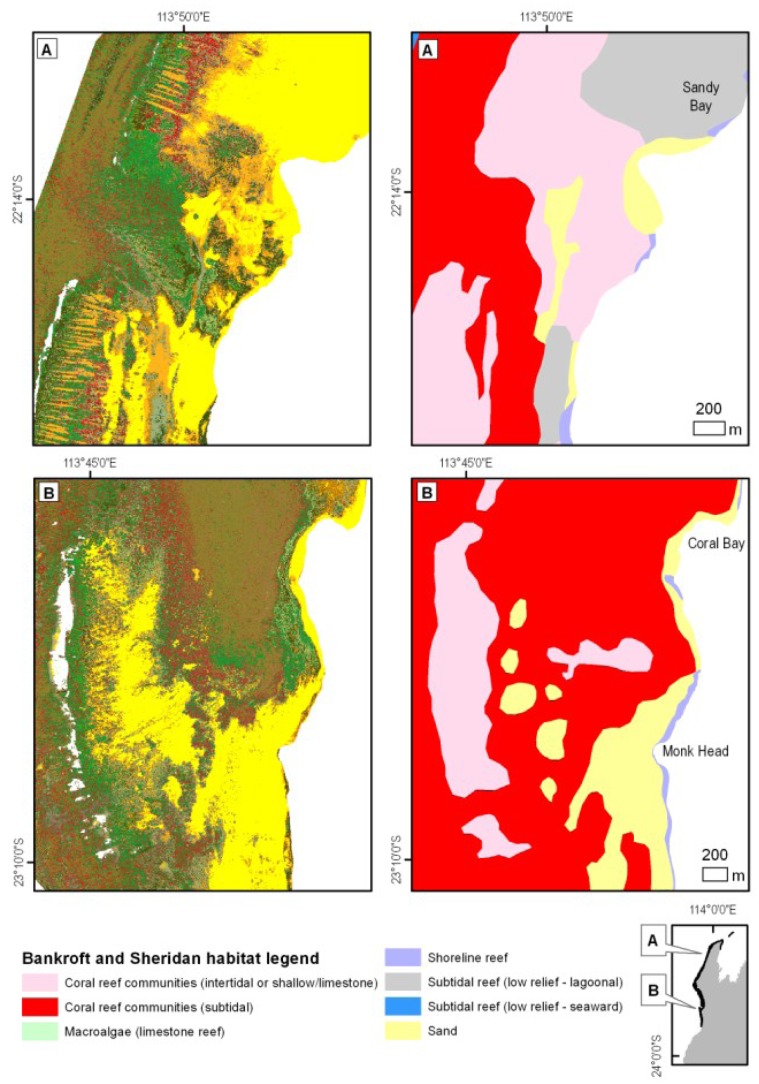
New hyperspectrally derived (left) and existing (Bancroft and Sheridan) [6], [7] (right) habitat maps for two selected areas in the northern (upper panels) and central regions (lower panels) of Ningaloo Reef. (A) Reef channel west of Sandy Bay is characterized by a mix of macro-algae and pavement in the new map, contrasting with mostly coral cover in the existing map [6], [7]. Spur and groove patterns are evident on the reef flats north and south of the channel, a feature which is missed in the panel on the right. (B) Coral communities around Coral Bay with distinct transitions between coral and macro-algae dominated cover as well as clusters of bommies adjacent to the sandy lagoons which are overrepresented in area in the existing map. Legend for the maps in the left hand panels is the same as on Figure 7.

Habitat maps created in this study were contrasted with those currently used by managers and researchers [6]. They showed an overall match between broad features such as sand, back reef coral habitat and macro-algae but had large discrepancies in the spatial extent as well as feature descriptions (Figure 14) and indicated much lower area covered by coral mosaics. Maps derived from the hyperspectral data captured finer spatial features such as spur and groove environments on the reef flats, clusters of bommies, alluvial fans, pavement ridges and transition zones between coral, algae and pavement substrates, features largely missing in the currently used maps [6]. The number and detail of information classes was also different, making pixel by pixel or polygon by polygon quantitative comparisons based on class names virtually impossible.

## Discussion

This study has successfully demonstrated the utility of high resolution airborne optical remote sensing to map shallow marine habitats across three degrees of latitude using operational methods. Data processing used in the study allowed for extraction of highly detailed marine habitat information as well as seamless bathymetry for waters down to 20 m depth. This detection limit is consistent with previous work on coral reefs elsewhere, e.g. [15], [21]. Corrections to the remote sensing data were implemented with operational methods using standardized parameters and processed on a stand-alone desktop computer. Hyperspectral data were successfully classified over four days on whole mosaics, enabling rapid implementation of a single classification approach for the whole study area. This method allows for future processing of additional data sets and comparisons over time for selected areas and is also suitable for processing multi- or hyperspectral satellite data [48], [49].

### Spectral Analysis

Benthic habitats of the Ningaloo Reef are highly diverse and mixed [8], even at the 3.5 m pixel scale of this study. While the *in sit*u spectra collection was limited to areas accessible to divers or shore sampling, it proved sufficient to inform early decisions on the direction of the classification scheme, including spectral separability between different reef components. Effort was made to cover different areas along the reef from the southern, narrow reef areas, through the central, wide lagoon areas with extensive coral cover, to the northern areas dominated by macro-algae and limestone pavement.

Creation of the spectral library allowed for determination of the degree of separability of the dominant spectral cover components. The selection of biotic or abiotic cover types for the final classification was largely based on frequency of occurrence in the field data set as well as spectral separability. Spectral analysis of the image spectra using the PCA and JM distance allowed for exact measures of separability to be determined, thus eliminating subjectivity in the final class selection. This approach provided a sound basis for refining or regrouping classes before the final classification which used bottom reflectance and the first two derivatives.

Field spectra were collected from a wide range of biotic and abiotic covers and represented dominant types, not spectral mixes which typically exist at the pixel level. Some authors [23], [26] have noted that field spectra often were unlikely to capture the full spectral variability of marine habitats since large variability (due to season, for example) exists even at species level. Results from the spectral separability analysis of the field spectra were very similar to previous studies on corals and macro-algae such as [10], [21], [56], [57]. One study has shown clear separation between field spectra of *Montipora* spp., *Porites* spp., macro-algae (*Chlorodesmis fastigata*) and sediments containing benthic micro-algae [56]. All the brown coloured corals measured at Ningaloo exhibited a characteristic reflectance feature at 570 nm, also reported in previous studies [14], [21]. In another study, an airborne CASI instrument with 1 m^2^ pixels and 10 broad spectral bands allowed for spectral separation between *Porites* spp., living *Pocillopora* spp., old and recently dead *Porites* spp. and *Pocillopora* spp. as well as the macro-alga *Halimeda* spp. and coralline red algae [22]. Depth of water and subsequent attenuation of the signal was, however, a major limitation in that particular study.

Nearly all discriminating spectral features in coral and macro-algae spectra occurred in narrow wavelength ranges, sometimes as broad as 20 nm, but often of the order of 10 nm [21]. Multispectral instruments cannot separate some of the information classes and this is where hyperspectral instruments have a definite advantage. Many living reef components share similar pigments and, therefore, the spectral separability of non-living components is often confounded by the presence of an epilithic algal film or turfing algae [23], [26]. While some studies have separated corals according to colour only [21], we additionally achieved splitting of corals into growth forms, such as branching, digitate and tabulate, as it was found that different texture, morphology and shadowing resulted in brightness differences.

Mapping macro-algae, turfing or coralline algae was not a priority for this project, however, a number of field spectra of commonly occurring species were collected. In the classification scheme, all algae were grouped on the basis of their percentage cover within a quadrat (pixel), rather than using species-specific data. This was mostly because, apart from *Sargassum* spp., all other algae, whether turfing, fleshy or coralline, occurred in highly mixed assemblages and at low cover. From the coastal management perspective, it often is very valuable to map algal communities in detail [4]. Such maps could facilitate better understanding and management as interactions of algae with coral communities are especially important in areas of periodic or chronic disturbance. With further fieldwork, the current hyperspectral data set for Ningaloo Reef could be reprocessed to enhance the level of description for algal communities. High accuracies and separation between canopy and turfing algae have been achieved using the same instrument and pre-processing approach in an area dominated by various algae and seagrasses around Rottnest Island, 900 km south of Ningaloo [58].

Although the spectral library results showed good separation between different macro-algae, turfing algae, live and dead corals, there were very few homogenous pixels in the airborne data to allow such classes to be included. Absence of extensive cover by recently dead coral was supported by the findings of long term monitoring investigations at Ningaloo [59], where only a small percentage of recently dead coral has been found in the Coral Bay area. Older dead coral specimens were all overgrown by macro-algae [59]. In another study on remote sensing detection of dead corals, bleached and non-bleached corals could only be mapped with pixels of about 0.01 m^2^ [38] and very high spectral resolution sensors were needed to separate spectra of some corals and macro-algae.

### Classification Approach

The hierarchical classification approach used in this study reflected typical, complex reef mosaics of coral, various algae, sand and pavement, and thus it was logical to classify the images first into basic biotic and abiotic components and then to further organise them at more detailed levels within these broad classes. This approach was similar to the scheme used by Harvey et al. [60] in the temperate areas at Rottnest Island, off the Western Australian coast. Using the same instrument, that study showed good separation between seagrasses, canopy and turfing algae and abiotic components of the marine benthos [58], [60]. In other regions, a similar approach using hyperspectral data produced satisfactory results with only field derived spectra [61], [62], although with fewer spectral end members.

Spectral analysis of the image derived spectra prior to image classification allowed for refinements in final class definitions, for example, classes with the same biotic or abiotic components requiring merging. While some classes (e.g., “blue-tip” branching *Acropora*) were very different from other branching corals, a number of classes containing a low percentage of coral and more than 50% of macro-algae were spectrally very similar. The high spectral resolution of the sensor also highlighted variability in spectral properties of abiotic cover types such as sand and pavement from the northern to the southern extent of the study area. Large spectral variability of abiotic components was mostly due to grain sizes and mineralogical composition which varies along the coast [8].

The habitat classifications generated during this study fit into the more complex habitat classifications described by [4]. Hierarchical design of the classification developed here was driven primarily by the end-user needs (for example, managers requiring only medium level of detail but coral scientists needing more detailed information). This hierarchy, of course, also reflects the uncertainty in the classification and its subsequent accuracy. The advantage of the hierarchical arrangement method used here was that it was based on operational processing of remotely sensed data and standard, quadrat-based fieldwork, both easily reproducible and quantitative. Class names incorporated both description and percentage cover, hence, any future changes in percentage cover beyond ±10% are going to be measurable and thus important to monitoring of reef condition. In addition, the classification scheme developed here captured gradients of various biotic assemblages, in particular, at thematic levels 3 and 4.

A hierarchical design with a look-up table for the final habitat maps allows users to create their own maps specific to their needs. This approach also accommodates the fact that the definitions of habitats always have some arbitrary component in class labeling [4]. Presence of both qualitative and quantitative descriptions for the class labels goes some way towards ensuring that these maps can be interpreted easily, are unambiguous, and reflect the quantitative and qualitative characteristics of the habitats captured through the fieldwork and spectral analysis.

Many habitat classification schemes based on reef geomorphology have been developed and there appears to be a lot of consistency and standardisation [8], [63], [64]. In a study of reefs of the Florida Keys, 22 thematic classes with descriptive labels were used [65]. Some of these biotic components such as seagrasses were given an additional descriptor indicating abiotic component, for example: “seagrass on lime mud” and “seagrass on carbonate sand”. This was similar to the system used in the current study, although less complex. Some studies have separated corals according to colour only; this study additionally classified often similarly coloured corals based on growth form such as branching, digitate and tabulate which provides a lot more valuable baseline [21].

A number of studies have mapped coral cover using a semi-quantitative approach and described the coral cover as “low”, “medium” and “high” density [66], [67]. Other studies provided percentage density (intervals) for coral cover [68]. This is similar to the current study and probably the most realistic if the area of study is large and quite diverse. A study of reefs at Pacific Ocean islands, created 10 coarse and 56 detailed classes which incorporated information on depth, exposure, percentage cover of algae, coral and seagrasses, taxonomy and geomorphology [69]. It reported accuracy of mapping as greater than 75%. A multi-temporal study of Florida Keys with Landsat TM, examined community shifts over time in very broad terms, from coral to algal dominated [37]. Studies so far have used a mix of classification approaches and data sets, including multi-temporal data and have enabled biologists to study shifts in coral communities in space and time. What is crucial is that the logic of the classification scheme allows for comparisons to be made between locations and over time.

The choice of the classification approach is always an important one as the conventional “hard” spectral classification schemes are problematic when applied to mixed pixels because each pixel must be assigned to a single habitat class [70]. With the fuzzy logic approach used in this study, classes with equally high probabilities could be analysed and revised.

Previous studies have used a linear unmixing approach (assumes reflectance of the pixel to have a linear relationship to the sum of the end-member spectra) [26], [61], [62], [71], [72]. The limitation of this approach in mapping large areas is the need for a comprehensive field-derived spectral library of the marine cover components, possibly not practical for an area as large as Ningaloo Reef. As previously mentioned, many studies have shown that large spectral variability exists even for the same species [23], [26]. In addition, spectral mixing may not follow a simple linear model [26], [73]. Therefore, the spectral unmixing approach was not chosen due to insufficient number of field spectra covering the range of possible reef components encountered in the field. At a practical level, there was a substantial time constraint in that activity (diving and boating) and while we could have pursued collection of additional spectra, this would have been potentially intrusive (*in situ* extraction of reef biota in a marine protected area). Further, there were possible effects on the spectral behaviour of corals and algae during exposure to the air in order to follow the approach of creating representative mixed classes [26].

Results of the accuracy assessment, while ranging from 64% for the most detailed data set to 84% for the medium detail maps, were in the range of accuracies reported in similar studies elsewhere. Other studies using multi- and hyperspectral sensors which classified habitats to at least eight classes, all reported overall accuracies above 70% [33], [58], [74]–[76]. Lower accuracies in some classes can be attributed primarily to the spectral similarity between coral types and between coral and algal types as a result of similar reflectance and absorption features. This was particularly the case with patchy and sparse distributions of macro-algae.

Current airborne or satellite systems do not yet offer spatial or spectral resolutions to map coral reef communities at the species level [56] and further work is needed in understanding spectral separability of the different benthic components [21]. It has also been suggested that spectral variation of benthic classes limits the extent of remote sensing applications in mapping projects [73]. Spectral interactions between spectrally mixed substrates are far more complex than their terrestrial equivalents and more research is needed to allow integrations of remote sensing research into studies on coral health, condition and process monitoring [56]. Currently, operational projects, at regional-scale, including change mapping, rely on multispectral satellites such as Landsat TM, QuickBird, SPOT or more recently WorldView2 [48], [49], [63], [66], [68], [77]–[79]. Approach used in this study can be applied to such multispectral satellite data which are lower in cost [48], [49].

Comparison with the existing habitat maps for Ningaloo Marine Park [6] showed large discrepancies in the level of detail (thematic and spatial) which is not surprising as the methods of mapping, image interpretation and classification systems were quite different. The usual approach in remote sensing or GIS is to undertake quantitative analysis using pixel by pixel or polygon by polygon comparison. This was not sensible due to differences in definitions and high level of aggregation/generalisation on the existing map [6]. For example, the class “Coral reef communities (intertidal or shallow/limestone)” in [6], corresponds on our map to a mix of patchy limestone pavement and sand and hard coral (>90% cover), continuous pavement, continuous macro-algae, sparse turfing algae, patchy limestone pavement, sand and rubble and sparse hard coral. Because the proportions of the mix of our new classes changes across different geographic areas of the reef, it was not possible to develop consistent rules to amalgamate some of our classes to create the direct equivalence to the classes depicted on the existing map [6]. Remote sensing practitioners are very familiar with this problem. Robust, spatial quantitative methods for such comparisons exist, but the difficulty in this case is also at the definition or the thematic level for the data sets.

The two main advantages of using optical remote sensing for this study have been, firstly, the ability to seamlessly map marine habitats across a very large area using a single classification system and secondly, to extract seamless bathymetry (not presented here) across the whole system of lagoons, including very shallow waters over coral normally inaccessible to acoustic surveys. The clear, shallow waters along the Ningaloo coast naturally lend themselves to such optical remote sensing methods.

Findings from this study can be used for management and monitoring. A number of possible indicators include cover of corals, macro-algae, sand, limestone or rubble. Some of the past studies which mapped large scale reef systems focused on geomorphic [67] or biological aspects [12], [80]. Digital data sets and maps, such as those created during this study are effective tools to help understanding past [37], [65] and current distributions of reefs [8]. The ability to visualise the reef settings, patterns of distributions and degree to which different parts of the reef are connected are vital in designing ecological surveys, monitoring programs and for modeling studies. Future work could examine, in more detail, spatial patterns, distribution along the reef and relative sizes of particular benthic cover subsets. This could be undertaken in combination with bathymetry and its derivatives generated by this study. Additional data sets such as exposure to prevailing winds and currents, turbidity, position in relation to major bathymetric features (channel passes, slopes, and flat-bottomed lagoons) and geomorphology could aid in understanding of the distribution of biota. Recent studies have demonstrated the utility of habitat maps as surrogates of biodiversity in conservation planning [69] and the forthcoming, 2015 review of the management plan for Ningaloo Marine Park provides an opportunity to test it with the new high resolution maps created by this study [81].

### Summary and Conclusions

Effective management and monitoring of large marine protected areas require detailed data on distribution of benthic habitats. Large areas with complex bathymetry and very clear waters such as at Ningaloo Reef are highly suitable to the application of optical remote sensing as a means of gathering such data. Analysis techniques involved spectral analyses on *in situ,* as well as image-derived spectra. This facilitated the selection of classes, a fuzzy logic approach to generate probability images for each habitat class, and enabled the analysis of classes with equally high probabilities. It also allowed for development of the semi-automated supervised classification approach based on underwater visual census of benthic cover.

The outputs of image analysis contained final classification categories as well as per-pixel probability layers and overall percent cover of corals, macro-algae and sediment. Reef components were classified into abiotic and biotic, and then split further into sand, limestone pavement, several coral cover categories and macro-algae dominated classes. These were organised through a look-up table into five thematic information class levels.

This work demonstrated that it is possible to consistently map coral reef habitats over large areas (spanning three degrees of latitude) with a single processing rule set. We were also able to utilize a hyperspectral sensor to map different coral forms. With the use of a hierarchical classification scheme we offer greater choices in viewing the data, aiming to improve uptake of such data sets for management and monitoring. We have also demonstrated that hyperspectral remote sensing is well suited for automated mapping tasks. These baseline data can be used for ongoing and future monitoring programs using the same or simpler satellite-based multispectral sensors such as QuickBird or WorldView2 to detect change over areas of interest. Hyperspectral sensors provide a non-invasive and cost-effective approach to mapping and monitoring the extent and condition of reefs over large areas because of their capability to identify reef components on the basis of their spectral response.

## Supporting Information

Figure S1 **Overview of the airborne data image pre-processing used for the Ningaloo Reef study.**
(TIF)

Figure S2 **Workflow for processing of Ningaloo field data (spectra and percentage cover) to develop the classification system including training and validation data sets.**
(TIF)

Table S1 (DOCX)

Table S2 (DOCX)
