# Supplementary figures and images for: Ningaloo Reef: Shallow Marine Habitats Mapped Using a Hyperspectral Sensor
Source: PLoS One. 2013 Jul 26;8(7):e70105. doi: 10.1371/journal.pone.0070105 (PMC3724944; doi:10.1371/journal.pone.0070105)

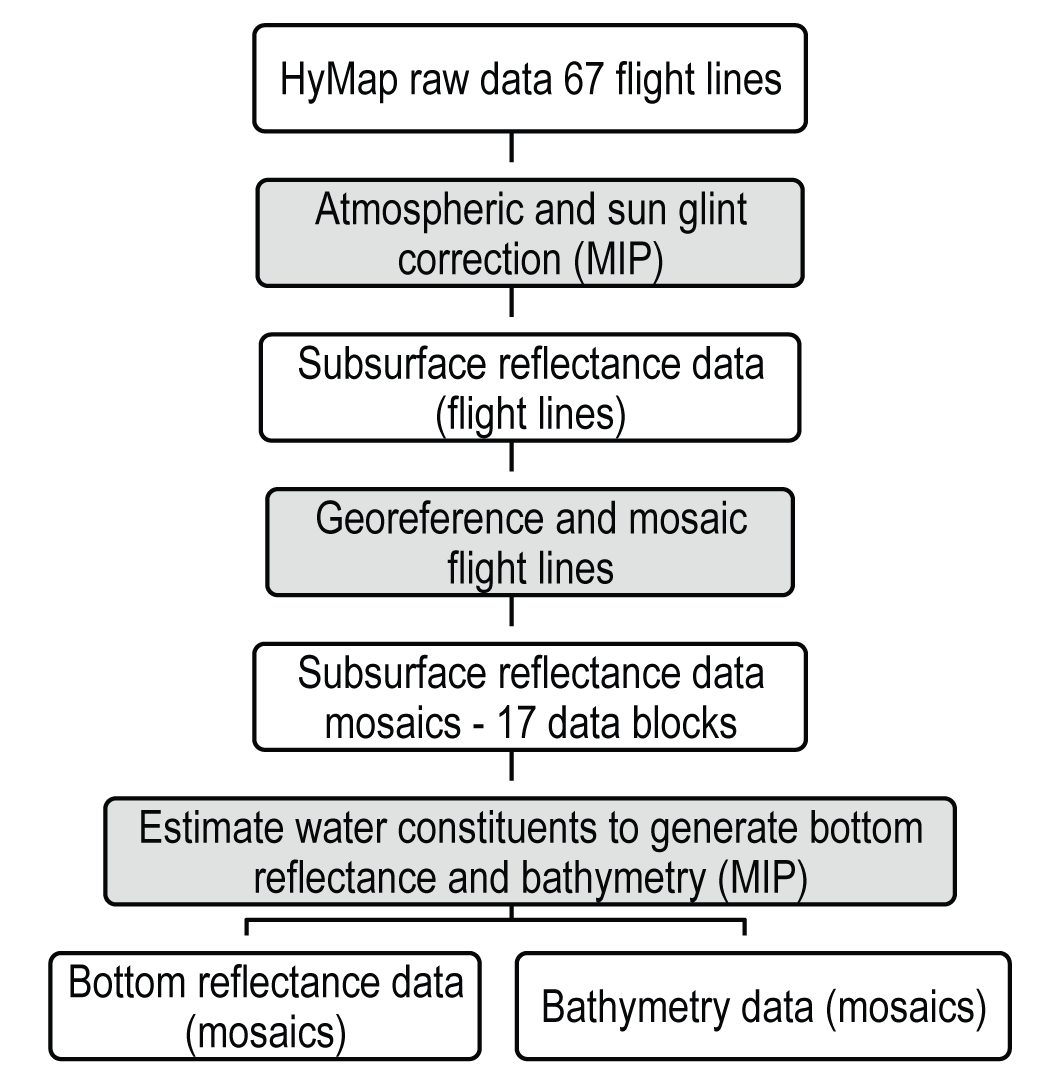

Supplement: Figure S1 — Overview of the airborne data image pre-processing used for the Ningaloo Reef study. (TIF) [file pone.0070105.s001.tif]

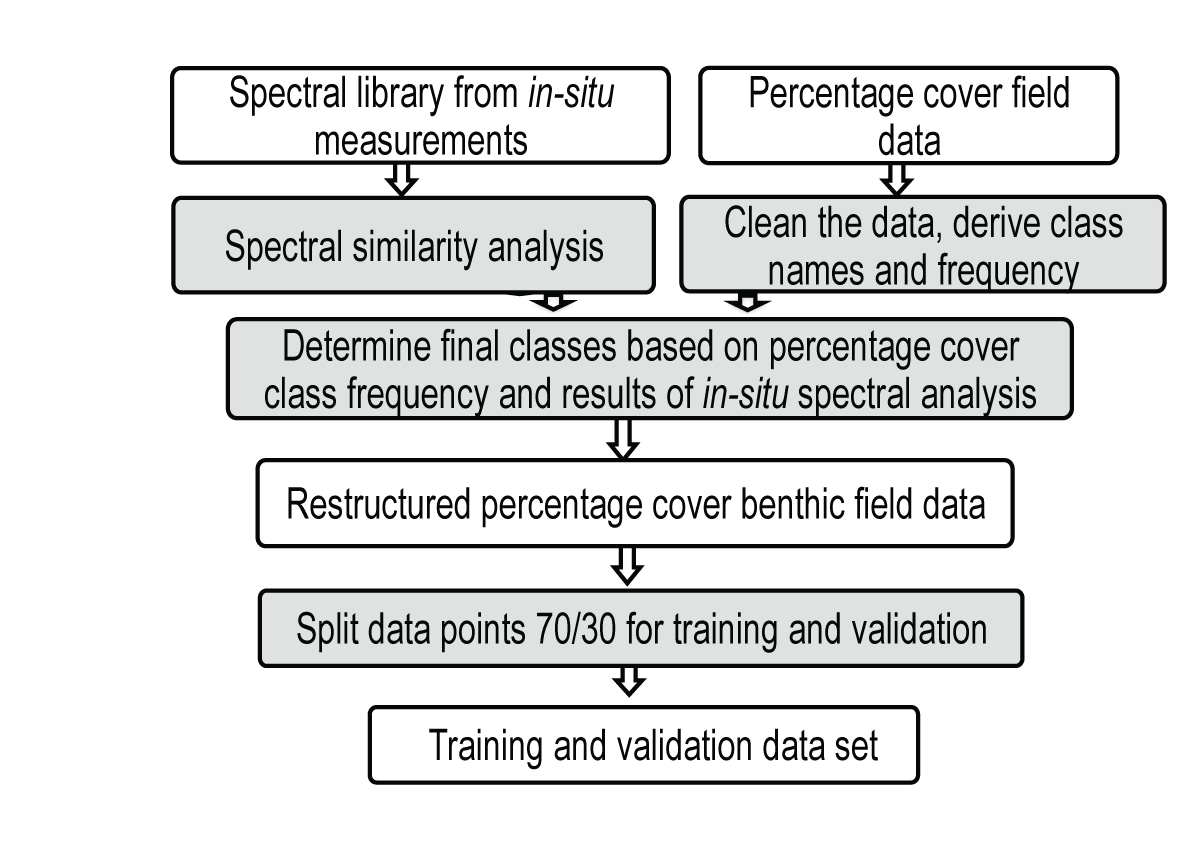

Supplement: Figure S2 — Workflow for processing of Ningaloo field data (spectra and percentage cover) to develop the classification system including training and validation data sets. (TIF) [file pone.0070105.s002.tif]
