# Supplementary material for: Ningaloo Reef: Shallow Marine Habitats Mapped Using a Hyperspectral Sensor
Source: PLoS One. 2013 Jul 26;8(7):e70105. doi: 10.1371/journal.pone.0070105 (PMC3724944; doi:10.1371/journal.pone.0070105)
Supplement: Table S1 — (DOCX) [file pone.0070105.s003.docx]

Table S1. Look-up table for marine habitat maps at different levels of detail for biotic and abiotic descriptors and degree of cover at Ningaloo Reef. Data have been sorted by Level 2a information to list description of class name for coral classes first.

| **Id** | **Level 1** | **Level 2a** | **Level 2b** | **Level 3a** | **Level 3b** | **Level 4a** | **Level 4b** | **Level 5** |
| --- | --- | --- | --- | --- | --- | --- | --- | --- |
| 3 | Mixed | HC | HC with TA- or MA-covered IDC or R | Dominant HC | Dominant HC with TA- or MA-covered IDC or R | Dominant HC with TA- or MA-covered IDC or R (HC=Continuous CT) | HC (65-90%) with TA- or MA-covered IDC or R (10-35%) (HC consists of CT (>90%)) | Continuous CT |
| 4 | Mixed | HC | HC with LP | Dominant HC | Dominant HC with LP | Dominant HC with LP (HC=Dominant CT with CD, CE and CS) | HC (65-90%) with LP (10-35%) (HC consists of CT (50-85%) with CD (5-20%), CE (5-20%) and CS (5-20%)) | Dominant CT with CD, CE and CS |
| 5 | Mixed | HC | HC with S | Dominant HC | Dominant HC with S | Dominant HC with S (HC=Continuous CBT) | HC (65-90%) with S (10-35%) (HC consists of CBT (>90%)) | Continuous CBT |
| 6 | Mixed | HC | HC with LP | Sparse HC | Sparse HC with LP | Sparse HC with LP (HC=Patchy CD and CT) | HC (10-35%) with LP (65-90%) (HC consists of CT (35-60%) with CD (35-60%)) | Patchy CD and CT |
| 15 | Mixed | HC | HC with TA- or MA-covered IDC or R | Patchy HC | Patchy HC with TA- or MA-covered IDC or R | Patchy HC with TA- or MA-covered IDC or R (HC=Continuous CD) | HC (35-60%) with TA- or MA-covered IDC or R (35-60%) (HC consists of CD (>90%)) | Continuous CD |
| 16 | Mixed | HC | HC with S | Patchy HC | Patchy HC with S | Patchy HC with S (HC=Continuous CT) | HC (35-60%) with S (35-60%) (HC consists of CT>90%) | Continuous CT |
| 18 | Mixed | HC | HC with TA- or MA-covered IDC or R | Patchy HC | Patchy HC with TA- or MA-covered IDC or R | Patchy HC with TA- or MA-covered IDC or R (HC=Continuous CT) | HC (35-60%) with TA- or MA-covered IDC or R (35-60%) (HC consists of CT (>90%)) | Continuous CT |
| 20 | Mixed | HC | HC with TA- or MA-covered IDC or R and S | Patchy HC | Patchy HC with TA- or MA-covered IDC or R and S | Patchy HC with TA- or MA-covered IDC or R and S (HC=Dominant CBT with sparse CD) | HC (20-45%) and TA- or MA-covered IDC or R (20-45%) with S (20-45%) (HC consists of CBT (65-90%) with CD (10-35%)) | Dominant CBT with sparse CD |
| 21 | Mixed | HC | HC with TA- or MA-covered IDC or R and S | Patchy HC | Patchy HC with TA- or MA-covered IDC or R and S | Patchy HC with TA- or MA-covered IDC or R and S (HC=Continuous CBT) | HC (20-45%) and TA- or MA-covered IDC or R (20-45%) with S (20-45%) (HC consists of CBT (>90%)) | Continuous CBT |
| 22 | Mixed | HC | HC with LP | Patchy HC | Patchy HC with LP | Patchy HC with LP (HC=Dominant CT with sparse CD) | HC (35-60%) with LP (35-60%) (HC consists of CT (65-90%) with CD (10-35%)) | Dominant CT with sparse CD |
| 23 | Mixed | HC | HC with LP | Patchy HC | Patchy HC with LP | Patchy HC with LP (HC=Continuous CD) | HC (35-60%) with LP (35-60%) (HC consists of CD (>90%)) | Continuous CD |
| 24 | Mixed | HC | HC with LP | Patchy HC | Patchy HC with LP | Patchy HC with LP (HC=Continuous CT) | HC (35-60%) with LP (35-60%) (HC consists of CT (>90%)) | Continuous CT |
| 25 | Mixed | HC | HC with S | Patchy HC | Patchy HC with S | Patchy HC with S (HC=Patchy CB, CT, CF, CM and CS) | HC (35-60%) with S (35-60%) (HC consists of CB (5-35%), CT (5-35%), CF (5-35%), CM (5-35%), CS (5-35%)) | Patchy CB, CT, CF, CM and CS |
| 26 | Mixed | HC | HC with TA- or MA-covered IDC or R | Patchy HC | Patchy HC with TA- or MA-covered IDC or R | Patchy HC with TA- or MA-covered IDC or R (HC=Patchy CD and CT) | HC (35-60%) with TA- or MA-covered IDC or R (35-60%) (HC consists of CD (35-60%) with CT (35-60%)) | Patchy CD and CT |
| 27 | Mixed | HC | HC with TA- or MA-covered IDC or R | Patchy HC | Patchy HC with TA- or MA-covered IDC or R | Patchy HC with TA- or MA-covered IDC or R (HC=Continuous CBT) | HC (35-60%) with TA- or MA-covered IDC or R (35-60%) (HC consists of CBT (>90%)) | Continuous CBT |
| 28 | Mixed | HC | HC with TA- or MA-covered IDC or R | Patchy HC | Patchy HC with TA- or MA-covered IDC or R | Patchy HC with TA- or MA-covered IDC or R (HC=Continuous CM) | HC (35-60%) with TA- or MA-covered IDC or R (35-60%) (HC consists of CM (>90%)) | Continuous CM |
| 37 | Biotic | HC | HC | Continuous HC | Continuous HC | Continuous HC (HC=Continuous CBT) | HC (HC consists of CBT (>90%)) | Continuous CBT |
| 38 | Biotic | HC | HC | Continuous HC | Continuous HC | Continuous HC (HC=Continuous CF) | HC (HC consists of CF (>90%)) | Continuous CF |
| 40 | Biotic | HC | HC | Continuous HC | Continuous HC | Continuous HC (HC=Dominant CT with sparse CD) | HC (HC consists of CT (65-90%) with CD (10-35%)) | Dominant CT with sparse CD |
| 41 | Biotic | HC | HC | Continuous HC | Continuous HC | Continuous HC (HC=Dominant CT with sparse CM and CS) | HC (HC consists of CT (55-90%) with CM (5-25%) and CS (5-25%)) | Dominant CT with sparse CM and CS |
| 42 | Biotic | HC | HC | Continuous HC | Continuous HC | Continuous HC (HC=Continuous CB) | HC (HC consists of CB (>90%)) | Continuous CB |
| 43 | Biotic | HC | HC | Continuous HC | Continuous HC | Continuous HC (HC=Continuous CD) | HC (HC consists of CD (>90%)) | Continuous CD |
| 44 | Biotic | HC | HC | Continuous HC | Continuous HC | Continuous HC (HC=Continuous CT) | HC (HC consists of CT (>90%)) | Continuous CT |
| 45 | Biotic | HC | HC | Continuous HC | Continuous HC | Continuous HC (HC=Continuous CM) | HC (HC consists of CM (>90%)) | Continuous CM |
| 7 | Mixed | HC, MA | HC and MA with LP and S | Sparse HC and MA | Sparse HC and MA with LP and S | Sparse HC and MA with LP and S (HC=Continuous CD) | HC (5-20%) and MA (5-20%) with LP (50-85%) and S (5-20%) (HC consists of CD (>90%)) | Continuous CD |
| 8 | Mixed | HC, MA, TA | HC, MA and TA with LP | Sparse HC, MA and TA | Sparse HC, MA and TA with LP | Sparse HC, MA and TA with LP (HC=Continuous CD) | HC (5-20%), MA (5-20%) and TA (5-20%) with LP (50-85%) (HC consists of CD (>90%)) | Continuous CD |
| 34 | Abiotic | LP dark | LP dark | Continuous LP dark | Continuous LP dark | Continuous LP dark | LP (>90%) | No HC or SC |
| 33 | Abiotic | LP light | LP light | Continuous LP light | Continuous LP light | Continuous LP light | LP (>90%) | No HC or SC |
| 17 | Abiotic | LP, R, S | LP, R and S | Patchy LP, R and S | Patchy LP, R and S | Patchy LP, R and S | LP (20-45%) with S (20-45%) and R (20-45%) | No HC or SC |
| 1 | Abiotic | LP, S | LP with S | Dominant LP with S | Dominant LP with S | Dominant LP with S | LP (65-90%) with S (10-35%) | No HC or SC |
| 19 | Abiotic | LP, S | LP with S | Patchy LP and S | Patchy LP with S | Patchy LP with S | LP (35-60%) with S (35-60%) | No HC or SC |
| 2 | Mixed | MA | MA with S | Sparse MA | Sparse MA with S | Sparse MA with S | MA (10-35%) with S (65-90%) | No HC or SC |
| 9 | Mixed | MA | MA with LP | Sparse MA | Sparse MA with LP | Sparse MA with LP | MA (10-35%) with LP (65-90%) | No HC or SC |
| 10 | Mixed | MA | MA with LP and S | Sparse MA | Sparse MA with LP and S | Sparse MA with LP and S | MA (5-20%) with LP (50-90%) and S (5-20%) | No HC or SC |
| 12 | Mixed | MA | MA with LP | Dominant MA | Dominant MA with LP | Dominant MA with LP | MA (65-90%) with LP (10-35%) | No HC or SC |
| 13 | Mixed | MA | MA with R | Dominant MA | Dominant MA with R | Dominant MA with R | MA (65-90%) with R (10-35%) | No HC or SC |
| 14 | Mixed | MA | MA with S | Dominant MA | Dominant MA with S | Dominant MA with S | MA (65-90%) with S (10-35%) | No HC or SC |
| 30 | Mixed | MA | MA with LP | Patchy MA | Patchy MA with LP | Patchy MA with LP | MA (35-60%) with LP (35-60%) | No HC or SC |
| 31 | Mixed | MA | MA with LP and S | Patchy MA | Patchy MA with LP and S | Patchy MA with LP and S | MA (20-45%) with LP (20-45%) and S (20-45%) | No HC or SC |
| 32 | Mixed | MA | MA with S | Patchy MA | Patchy MA with S | Patchy MA with S | MA (35-60%) with S (35-60%) | No HC or SC |
| 46 | Biotic | MA | MA | Continuous MA | Continuous MA | Continuous MA | MA (>90%) | No HC or SC |
| 29 | Abiotic | S | S | Continuous S | Continuous S | Continuous S | S (>90%) | No HC or SC |
| 35 | Biotic | SC | SC | Continuous SC | Continuous SC | Continuous SC | SC (>90%) | Continuous SC |
| 39 | Mixed | SC, HC | SC, HC with LP | Dominant SC, sparse HC | Dominant SC, sparse HC with LP | Dominant SC, sparse HC with LP (HC=Continuous CD) | SC (55-90%) and HC (5-25%) with LP (5-25%) (HC consists of CD (>90%)) | Dominant SC, Continuous CD |
| 36 | Mixed | SC, HC, MA | SC, HC and MA with LP | Dominant SC, sparse HC and MA | Dominant SC, sparse HC and MA with LP | Dominant SC, sparse HC and MA with LP (HC=Continuous CD or Patchy CD and CT) | SC (50-85%), HC (5-20%) and MA (5-20%) with LP (5-20%) (HC consists of CD (>90%), or CD (35-60%) with CT (35-60%)) | Dominant SC, Continuous CD or patchy CD and CT |
| 11 | Mixed | TA | TA with LP | Sparse TA | Sparse TA with LP | Sparse TA with LP | TA (10-35%) with LP (65-90%) | No HC or SC |
| 0 | Unclassified | Unclassified | Unclassified | Unclassified | Unclassified | Unclassified | Unclassified | Unclassified |
