# Supplementary material for: Ningaloo Reef: Shallow Marine Habitats Mapped Using a Hyperspectral Sensor
Source: PLoS One. 2013 Jul 26;8(7):e70105. doi: 10.1371/journal.pone.0070105 (PMC3724944; doi:10.1371/journal.pone.0070105)
Supplement: Table S2 — (DOCX) [file pone.0070105.s004.docx]

Table S2. Confusion matrix of accuracy assessment for level 4a (high number of fuzzy classes). The rows show the validation points, while the columns show the image derived labels. The value in the bottom right cell of each column is the sum of the diagonal. Class numbers are explained in Table S1.

|  | 1 | 3 | 4 | 5 | 7 | 12 | 16 | 20 | 29 | 35 | 37 | 40 | 42 | ∑ |
| --- | --- | --- | --- | --- | --- | --- | --- | --- | --- | --- | --- | --- | --- | --- |
| **1** | 36.19 | 0.00 | 0.00 | 0.00 | 0.00 | 0.00 | 0.00 | 0.00 | 0.00 | 0.00 | 0.00 | 0.00 | 0.00 | **36.19** |
| **3** | 3.81 | 1.90 | 0.00 | 0.00 | 0.00 | 0.95 | 0.00 | 0.00 | 0.00 | 0.00 | 0.00 | 0.00 | 0.00 | **6.67** |
| **4** | 5.71 | 0.00 | 3.81 | 0.00 | 0.00 | 0.00 | 0.00 | 0.00 | 0.00 | 0.00 | 0.00 | 0.00 | 0.00 | **9.52** |
| **5** | 0.95 | 0.00 | 0.00 | 0.95 | 0.00 | 0.00 | 0.00 | 0.00 | 0.00 | 0.00 | 0.00 | 0.00 | 0.00 | **1.90** |
| **7** | 1.90 | 0.00 | 0.00 | 0.00 | 2.86 | 0.00 | 0.00 | 0.00 | 0.00 | 0.00 | 0.00 | 0.00 | 0.00 | **4.76** |
| **12** | 0.95 | 0.00 | 0.00 | 0.00 | 0.00 | 7.62 | 0.00 | 0.00 | 0.00 | 0.00 | 0.00 | 0.00 | 0.00 | **8.57** |
| **16** | 1.90 | 0.00 | 0.00 | 0.00 | 0.00 | 1.90 | 0.00 | 0.00 | 0.00 | 0.00 | 0.00 | 0.00 | 0.00 | **3.81** |
| **20** | 0.95 | 0.00 | 0.00 | 0.00 | 0.00 | 1.90 | 0.00 | 3.81 | 0.00 | 0.00 | 0.00 | 0.00 | 0.00 | **6.67** |
| **29** | 0.00 | 0.00 | 0.00 | 0.00 | 0.00 | 0.00 | 0.00 | 0.00 | 3.81 | 0.00 | 0.00 | 0.00 | 0.00 | **3.81** |
| **35** | 0.95 | 0.00 | 0.00 | 0.00 | 0.00 | 0.00 | 0.00 | 0.00 | 0.00 | 5.71 | 0.00 | 0.00 | 0.00 | **6.67** |
| **37** | 0.00 | 0.00 | 0.00 | 0.00 | 0.00 | 1.90 | 0.00 | 0.00 | 0.00 | 0.00 | 0.00 | 0.00 | 0.00 | **1.90** |
| **40** | 0.95 | 0.00 | 0.00 | 0.00 | 0.00 | 0.95 | 0.00 | 0.00 | 0.00 | 0.00 | 0.00 | 3.81 | 0.00 | **5.71** |
| **42** | 3.81 | 0.00 | 0.00 | 0.00 | 0.00 | 0.00 | 0.00 | 0.00 | 0.00 | 0.00 | 0.00 | 0.00 | 0.00 | **3.81** |
| ∑ | **58.10** | **1.90** | **3.81** | **0.95** | **2.86** | **15.24** | **0.00** | **3.81** | **3.81** | **5.71** | **0.00** | **3.81** | **0.00** | **70.48** |
